# Association of reproductive risk factors and breast cancer molecular subtypes: a systematic review and meta-analysis

**DOI:** 10.1186/s12885-023-11049-0

**Published:** 2023-07-10

**Authors:** Xihua Mao, Chioma Omeogu, Shama Karanth, Ashwini Joshi, Clare Meernik, Lauren Wilson, Amy Clark, April Deveaux, Chunyan He, Tisha Johnson, Karen Barton, Samantha Kaplan, Tomi Akinyemiju

**Affiliations:** 1 Department of Epidemiology, College of Public Health, University of Kentucky, Lexington, KY USA; 2 Department of Population Health Sciences, School of Medicine, Duke University, Durham, NC USA; 3 UF Health Cancer Canter, University of Florida, Gainesville, FL USA; 4 The Cancer Prevention and Control Research Program, University of Kentucky Markey Cancer Center, Lexington, KY USA; 5 Department of Preventive Medicine and Environmental Health, College of Public Health, University of Kentucky, Lexington, KY USA; 6 Duke University Medical Center Library & Archives, Duke University School of Medicine, Durham, NC USA; 7 Duke Cancer Institute, Duke University, Durham, NC USA

**Keywords:** Breast cancer, Reproductive factors, Molecular subtypes, Hormone receptor, Meta-analysis

## Abstract

**Background:**

Associations between reproductive factors and breast cancer (BC) risk vary by molecular subtype (i.e., luminal A, luminal B, HER2, and triple negative/basal-like [TNBC]). In this systematic review and meta-analysis, we summarized the associations between reproductive factors and BC subtypes.

**Methods:**

Studies from 2000 to 2021 were included if BC subtype was examined in relation to one of 11 reproductive risk factors: age at menarche, age at menopause, age at first birth, menopausal status, parity, breastfeeding, oral contraceptive (OC) use, hormone replacement therapy (HRT), pregnancy, years since last birth and abortion. For each reproductive risk factor, BC subtype, and study design (case–control/cohort or case-case), random-effects models were used to estimate pooled relative risks and 95% confidence intervals.

**Results:**

A total of 75 studies met the inclusion criteria for systematic review. Among the case–control/cohort studies, later age at menarche and breastfeeding were consistently associated with decreased risk of BC across all subtypes, while later age at menopause, later age of first childbirth, and nulliparity/low parity were associated with increased risk of luminal A, luminal B, and HER2 subtypes. In the case-only analysis, compared to luminal A, postmenopausal status increased the risk of HER2 and TNBC. Associations were less consistent across subtypes for OC and HRT use.

**Conclusion:**

Identifying common risk factors across BC subtypes can enhance the tailoring of prevention strategies, and risk stratification models can benefit from subtype specificity. Adding breastfeeding status to current BC risk prediction models can enhance predictive ability, given the consistency of the associations across subtypes.

**Supplementary Information:**

The online version contains supplementary material available at 10.1186/s12885-023-11049-0.

## Introduction

Breast cancer (BC) is the most common cancer type and a leading cause of cancer death among women globally [1, 2]. Established risk factors for BC include genetic, reproductive, and lifestyle-related factors, contributing to variations in worldwide BC incidence rates [3]. Reproductive factors that have been linked with BC risk include age at menarche, age at menopause, menopausal status, pregnancy-related factors (age at first birth, parity, breastfeeding, years since last birth), oral contraceptive (OC) use, and hormone replacement therapy (HRT) [3–6]. These factors likely influence breast cancer risk through alterations in circulating levels of hormones such as estrogen [4]. Certain reproductive risk factors such as HRT and pregnancy may directly change hormonal levels, while other risk factors such as age at menarche and age at menopause are markers for the lifetime duration of hormonal exposures [5].

BC is recognized as a heterogeneous cancer due to differences in tumor and genomic features that indicate different etiology and prognosis [6]. Based on hormone receptors (estrogen (ER) and progesterone (PR)) and expression level of human epidermal growth factor receptor 2 (HER2), BC can be classified as luminal A (ER + and/or PR + , HER2 −), luminal B (ER + and/or PR + , HER2 +), HER 2-overexpression (ER − , PR − , and HER2 +), triple-negative, and basal-like (ER − , PR − , and HER2-),–luminal A being the most common of all the subtypes [7]. Although several studies have evaluated the associations between reproductive factors and molecular subtypes, results have been inconsistent [8–10]. A 2016 meta-analysis that evaluated 15 studies reported that parity was associated with decreased risk of luminal subtype and later age at first birth was associated with increased risk; while breastfeeding was associated with decreased risk of both luminal and triple-negative BC subtypes [6]. The meta-analysis included studies published up to 2014 and evaluated only three reproductive factors; age at first birth, parity, and breastfeeding. In the present systematic review and meta-analysis, we extend the review period through 2021 and examine 11 separate reproductive factors associated with each BC molecular subtype: age at menarche, age at menopause, age at first birth, menopausal status, parity, breastfeeding, OC use, HRT use, pregnancy, years since last birth, and abortion. The purpose of this study is to: i) comprehensively summarize the published literature on reproductive risk factors and molecular BC subtypes, and ii) generate summary estimates of the associations between reproductive risk factors and BC molecular subtypes.

## Methods

**Fig. 1:**
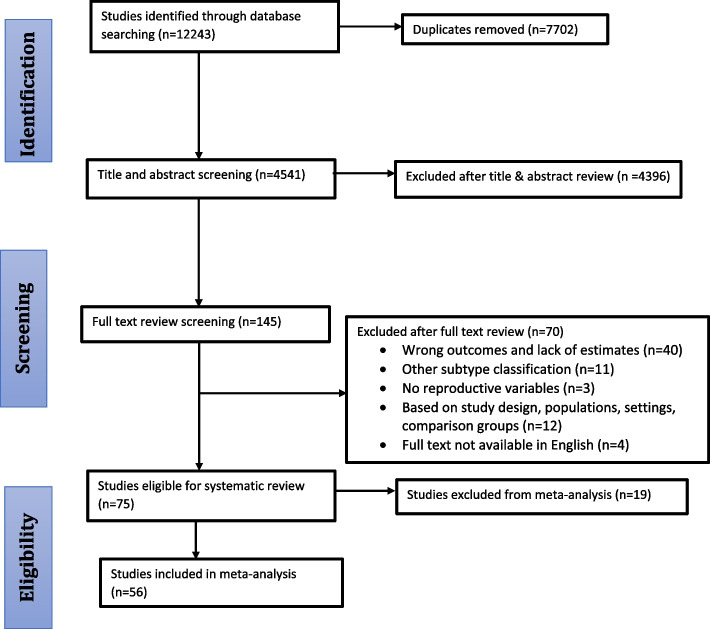
PRISMA flow chart for BC subtypes systematic review and meta-analysis

This systematic review and meta-analysis were conducted according to the Preferred Reporting Items for Systematic Reviews and Meta-Analyses (PRISMA) recommendations (Fig. 1) [11, 12]. Primary studies published in the English language between January 2000 and April 2021 were retrieved from PubMed, Scopus, and Embase. We focused on studies published after 2000 to account for the updated research based on breast tumor classifications and different subtypes. For instance, a study conducted by Perou et al., 2000 demonstrated how tumors can be categorized into different subtypes of breast cancer based on their unique patterns of gene expression, which may contribute to differential analysis of molecular gene expression patterns of BC tumors in studies conducted after 2000 [13]. The search strategy included MeSH and non-MeSH key terms for 1) “Breast Neoplasms”, and specific subtypes evaluated which included: luminal A, luminal B, HER-2- overexpression (HER2), basal-like, and triple-negative/basal-like (TNBC) breast neoplasms; 2)“Reproductive behavior” as well as specific reproductive factors, including menarche, abortion, parity (children born alive), breastfeeding, pregnancy (including live pregnancies and abortions), contraceptives, menopause, menstruation, menstrual period, age at first birth, birth control, birth intervals, and hormone replacement therapy. The specific search strategy is presented in the Supplementary materials.

### Study eligibility

Included studies focused on BC subtypes and reproductive factors of interest. Studies were excluded if they were published in languages other than English; full texts were not available; examined BC subtypes other than luminal A, luminal B, HER-2, TNBC, or outcomes other than BC subtypes; only analyzed non-reproductive risk factors or none of our a priori reproductive risk factors of interest; if effect estimates (i.e., odds ratios, relative risks, hazard ratios) were unavailable; or were study designs other than case–control, case-case, or cohort.

### Selection

Two authors (CO, SK) reviewed the titles, abstracts, and full text of all studies retrieved from electronic databases. Discrepancies in selection were resolved by consensus, and disagreements were resolved in consultation with a third author (XM). A total of 12,243 studies were retrieved from database searches. We excluded 7,702 duplicate articles, and 4,396 articles after the title and abstract review, resulting in 145 articles for full-text review. Of these, 40 studies were excluded due to non-relevant outcomes (e.g., percentages, proportions, means rather than OR or RR estimates); 11 studies due to other subtype classifications; 12 studies due to study design; 4 full texts were not available in English; and 3 studies lacked reproductive variables, resulting in 75 eligible studies. Of these, 56 studies reported estimates for the association between reproductive factors and BC subtypes and were included in meta-analysis (Fig. 1).

### Data extraction

Data was abstracted by one author (CO) and independently reviewed and verified based on original full texts by the two other authors (SK and AJ). Information on author, year, study design, study characteristics (country of study, sample size, data source, patient race, etc.), BC subtypes (luminal A, luminal B, HER-2, and TNBC), reproductive factors, covariates, and corresponding risk estimates and their 95% confidence intervals (CI) were recorded.

### Statistical analysis

Odds ratios (OR), relative risks (RR), and hazard ratios (HR) for reproductive factors comparing the most extreme (e.g., highest vs. lowest) categories were extracted. To ensure consistency in the meta-analysis, if needed, ratio measures were inverted to ensure reference categories matched across studies. Odds ratios and hazard ratios were converted to approximate RRs and 95% CIs. Studies were included in the meta-analysis when at least three studies of the same exposure-outcome combination were available. The meta-analysis was conducted separately for each combination of exposure and BC subtype. All 11 reproductive factors were included in the systematic review, but only eight that were examined in at least three studies were included in the meta-analysis: 1) age at menarche, 2) age at menopause, 3) age at first birth, 4) menopausal status, 5) parity, 6) breastfeeding, 7) OC use and 8) HRT use. Pregnancy, years since last birth, and abortion were qualitatively summarized. Study-level results are presented in supplemental materials (Supplementary Figs. 1-16). Random effects models were used to compute pooled estimates with 95% CIs for each reproductive factor and molecular subtype for both case–control/cohort, and case-case studies separately. We combined cohort and case–control studies together in our analysis based on similarity in results and strength of study design. In the case-case analysis, luminal A was the reference group as it was the most common subtype reported as a reference in the studies. The Q-statistic was used to assess the presence of between-study heterogeneity; the *I*^2^-statistic was used to examine the proportion of variation between studies due to heterogeneity (p-values reported with effect estimates correspond to the I^2^ *p*-value); [14] and the Egger test was used to assess publication bias [15]. Analyses were conducted using STATA version 15 (Stata Corp LLC, College Station, TX).

## Results

Seventy-five studies met the inclusion criteria and were deemed suitable for the systematic review (Fig. 1). The characteristics of included studies are summarized in Table 1 and Supplementary Table 1. Most eligible studies were published between 2011 and 2021 (*n* = 59), and about 40% of the studies were conducted in the United States. Of the seventy-five studies, thirteen were cohort studies, thirty-six studies were case–control, twenty-two were case-case studies, and four studies were both case–control and case-case study designs. Nineteen of the seventy-five studies reported data that could not be meta-analyzed, resulting in fifty-six studies included in meta-analysis. Four cohort studies [16–19] that reported hazard ratios to evaluate the association of reproductive factors and subtypes were combined with case–control studies in the meta-analysis.

**Table 1:** Study characteristics of included studies for systematic review and meta-analysis (*n*=75)

First Author, year	Study Design	Study Period	Sample size	Exposure	Outcome of interest	Estimates	Covariates
Abubakar, 2018 [20]	Case-case	2003–2016	3,012 cases	Menarche, parity, breastfeeding, age at first full pregnancy	Luminal B-like, HER2 enriched BC, TN BC	OR (95% CI)	Ethnicity, Menarche, Parity and BF, Age at FFP, Family History, BMI
Ambrosone, 2014 [21]	Case–control	2002–2008	917 (1,015 controls)	Breastfeeding, lactation, parity	TN BC	OR (95% CI)	Number of live births, breastfeeding, months breastfeeding, parity and lactation
Atkinson, 2016 [22]	Case–control	2004–2012	224 (396 controls)	Age at menarche, parity, age at first pregnancy, breastfeeding history, menopausal status	TN BC, HER2neu + BC, Luminal	OR (95% CI)	Age, age of menarche, parity, number of children, nulliparous, age at first pregnancy, breast-feeding history, body mass index, smoking history, menopausal status, breast cancer family history
Beaber, 2014 [23]	Case–control	2004–2010	985 cases (882 controls)	Lifetime duration of OC use, OC: current use vs. Never, Duration of OC use in the prior 5 years, time since last use, age at first use	ER-/HER2 + BC, TH BC, ER + , ER-	OR (95%CI)	Lifetime duration of OC use, OC: current use vs. Never, Duration of OC use in the prior 5 years, time since last use, age at first use
Benefield, 2021 [24]	Case–control and Case-case	1993–2013	2,354 cases (2,932 controls)	Age at menarche, age at first full-term birth, parity and breastfeeding, lifetime breastfeeding duration, OC use	ER-/HER2 + BC, Luminal B BC, Basal-like BC	OR (95%CI)	Age at menarche, age at first full-term birth, parity and breastfeeding, lifetime breastfeeding duration, OC use, BMI, WHR
Bethea, 2015 [25]	Case–Control	1995–2015	494 (10,044 controls)	OC use	TN BC and all other subtypes	OR (95% CI)	Never users, ever users, OC recency, OC duration, joint OC exposure
Brandao, 2021 [26]	Case–control	2015–2017	138 cases (638 controls)	Parity, number of live births	HR- BC, HR + BC, HR + /HER2-, HER2 + BC, TN BC	aOR (95%CI)	Parity, number of live births
Brouckaert, 2017 [27]	Case -control and Case-case	N/A	11, 328 cases	Age at menarche, Age at FFTP, Parity	Luminal B-like, Luminal HER2-like, HER2-like, TNBC, HER2 + BC	OR (95% CI)	Parity, age at menarche, age at FFTP
Cerne, 2012 [28]	Case control	2006–2008	784 (709 controls)	Age at Menarche, Full-term pregnancy, Breastfeeding, Age at first full-term pregnancy, Duration of OC use (years), Duration of HRT use (years)	HER2 + BC, HER2- BC	OR (95% CI)	Body mass index, age at menarche, full-term pregnancy, breastfeeding, age at first full-term pregnancy, infertility treatment, duration of OC use, Duration of HRT, Regimen of HRT, Number of mammograms, first-degree relative with breast and/or ovarian cancer, smoking, alcohol consumption
Chauhan R., 2020 [29]	Cohort	Jan – Dec 2019	446 cases	Menopausal status	Luminal A BC, Luminal B BC, Her2 overexpression BC, TN BC	N (%)	Menopausal status
Chen, 2016 [30]	Case-case	2004–2012	2,710 cases	Number of full-term pregnancies, Age at first full time pregnancy, Age at menopause, Ever breast fed a child, Breastfeeding duration,	Luminal B BC, TN BC, HER2-overexpressing BC	OR (95% CI)	Number of full-term pregnancies, Age at first full time pregnancy, Age at menopause, Age at menarche, Ever breast fed a child, Breastfeeding duration
Collins L.C, 2015 [31]	Cohort	2006-2015	707 cases	Parity, time since last pregnancy	Luminal A BC, Luminal B BC, HER2 overexpressing BC, TN BC	N (%)	Parity, time since last pregnancy
Cruz, 2013 [32]	Case-case	2007–2010	627 cases	Time since last full-term pregnancy	HER2 + BC	OR (95% CI)	Time since last full-term pregnancy
Cui, 2014 [33]	Case–control	2001–2011	2,510 cases	HRT use BMI < 25	Luminal A BC, Luminal B BC, HER2 overexpression BC, TN BC	OR (95% CI)	HRT use, Hysterectomy with/w/o bilateral oophorectomy
De Mulder, 2018 [34]	Cohort study	2000–2009	1,306 cases	Time since last pregnancy	Luminal A-like BC, Luminal B-like BC, Luminal HER-2 BC, HER-2-like BC	OR (95% CI)	Time since last pregnancy
Devi, 2012 [35]	Case-case	1998–2009	1,034 cases	Menopausal status, Age at menopause, Age at menarche, Parity, Average duration of breastfeeding (months)	HER2-overexpressing BC, TN BC	OR (95% CI)	Ethnicity, age at diagnosis, menopausal status, age at menopause, familial history, age at menarche, parity, average duration of breastfeeding (months), BMI, BMI premenopausal, BMI postmenopausal
Dogan, 2011 [36]	Case–control	N/A	500 cases	HRT, Lactation, Age at menopause, Age at menarche	Luminal B BC, HER + BC, TN BC	OR (95% CI)	HRT, Tumor size, BMI, IDC, ILC, ILC-ITC, Axillary involvement, Lactation, age at menopause, age at menarche, age
Dolle, 2009 [37]	Case–control study	1983–1992	1,286 cases	Age at menarche, Number of live births, Age of first birth, Lactation, Abortion, OC use, OC duration	HER2 + , HER-, TN BC	OR (95% CI)	Age, race, education, annual income, family history of breast cancer, BMI, smoking, alcohol use, age at menarche, number of live births, age at first birth, lactation, abortion, OC use, OC duration, age at first use, years since first use, years since last use
Ellingjord-Dale, 2017 [38]	Case–control	2006 to 2014	6,471 (32,355 controls)	Age at first birth, Number of pregnancies lasting 6 + mos, breastfeeding duration in parous women, menopausal status, age at menopause, age at start of OC use, duration of OC, age at start of intrauterine device, duration of intrauterine device yrs, HT use, duration of HT use yrs, duration of estrogen and progesterone use yrs	Luminal A BC, Luminal B like-HER2- BC, Luminal B like-HER2 + BC, HER2 overexpressing BC, TN BC	OR (95% CI)	Age at first birth, number of pregnancies lasting 6 + months, parous women duration of breast feeding (months), menopausal status, age at menopause, age at start of OC (years), duration of OC (years), age at start of intrauterine device (years), duration of intrauterine device (years), HT use, duration of HT (years), Duration of estrogen and progesterone therapy (years)
Fortner, 2019 [16]	Case-case	1976–2013	12,452 cases	Parity/breastfeeding	Luminal A BC, Luminal B BC, HER2-enriched BC, Basal-like BC	HR (95% CI)	Nulliparous, parous women never breastfed, parous women ever breastfed
Gaudet, 2011 [39]	Case–control	1980–1982	890 (3,432 controls)	Age at menarche, Nulliparous, Age at first birth per 5 years (among parous), Month of breastfeeding per 6 months (among parous), Ever use of OC	Luminal A BC, Luminal B BC, Her-2/neu + BC, TN BC	OR (95% CI)	Age at diagnosis, age at menarche, nulliparous, age at first birth per 5 years (among parous), months of breastfeeding per 6 months (among parous), BMI per WHO category (by menopausal status), benign breast disease, positive family history of breast cancer
Gaudet, 2018 [40]	Case-case	1990–2006	11,741 (606,025 controls)	Parity, Age at first live birth among parous women, age at menarche, age at menopause, ever use of OC	Luminal A BC, Luminal B BC, HER2-enriched BC, TN BC	HR (95% CI)	Parity, number of live births among parous women, age at live birth among parous women, age at menarche, years between menarche and first birth among parous women, age at menopause, ever use of oral contraceptives, first-degree family history of breast cancer, personal history of benign breast disease, BMI
Giudici, 2017 [41]	Case–control	2006–2014	286 (578 controls)	Age at menarche, OC use, breastfeeding duration	Luminal A BC, Luminal B BC	OR (95% CI)	Age at menarche, BMI, Oral contraceptive use, benign breast disease, family history of breast cancer, breastfeeding duration
Holm, 2017 [42]	Case–control	2011–2013	2,632 (15,945 controls)	Parity, age at first birth parous women only, Breastfeeding, parous women only, Breastfeeding including all women	Luminal A BC, Luminal B BC, HER2-overexpressing BC, Basal-like BC	OR (95% CI)	Parity, age at first birth, breastfeeding, breastfeeding all women
Horn, 2014 [43]	Case-case	1961–2008	21,532 cases	Age at menarche, Age at first birth, Nulliparous versus parous, Breastfeeding (ever versus never), Breastfeeding (total duration)	Luminal A, Luminal B (HER2-), Luminal B (HER2 +), HER 2 BC, Basal-like BC	HR (95% CI)	Age at menarche, Age at first birth, Nulliparous versus parous, Number of births among parous women, Breastfeeding (ever versus never), Breastfeeding (total duration)
Huang, 2000 [44]	Case–control	1993—1996	577 cases (790 controls)	Age at menarche, age at FFTP yrs, age at FFTP yrs, Abortion/Miscarriage, Breastfeeding, OC use, HRT	HER-2/neu + BC, HER-2/neu- BC	OR (95% CI)	Age at menarche, nulliparity/age at first full-term pregnancy, abortion or miscarriage, breastfeeding, oral contraceptive use, hormone replacement therapy, body mass index, waist:hip ratio, first degree family history of breast or ovarian cancer, medical radiation to the chest, alcohol drinking during most recent age range, smoking, education
Ihemelandu CU, 2007 [45]	Cohort	1998–2005	372 cases	Menopausal status	Luminal A BC, Luminal B BC, HER2-overexpressing BC, TN BC	N (%)	Menopausal status
Islam, 2012 [46]	Case–control	2003–2005	706 cases (1,412 controls)	Age at menarche, parity, Age at first live birth years, Breastfeeding history, age at menopause	HER2-overexpressing BC, TN BC	OR (95% CI)	Age at menarche, parity, age at first live birth, breastfeeding history, age at menopause
Jeong S.H., 2017 [47]	Case–control	2004–2012	25,778 cases (101,017 controls)	Number of children, Breastfeeding duration	Luminal A BC, Luminal B BC, HER2-overexpressing BC, TN BC	OR (95%CI)	Parity, number of childbirths, breastfeeding, breastfeeding duration
John, 2018 [48]	Case–control	1995–2009	558 cases (5,111 controls)	Age at menarche, Parity by history of breastfeeding, Parity (number of FTPs), Duration of breast-feeding (months), Parity by breast-feeding duration (months)	TN BC	OR (95% CI)	Age at menarche, parity by history of breastfeeding, parity (number of FTPs), duration of breastfeeding (months), parity by breast-feeding duration (months)
Kwan, 2009 [49]	Case-case	1997–2008	2,544 invasive BC cases	Menopausal status, Age at first full-term pregnancy yrs, parity, Lifetime duration of breastfeeding, Parity among never breastfed, Parity among > = 4 mos breastfed, HRT (postmenopausal only), OC use	Luminal B BC, HER2-overexpressing BC, TN BC	OR (95% CI)	Age at diagnosis, race/ethnicity, menopausal status, family history, age at first full-term pregnancy, parity, lifetime duration of breastfeeding, parity among breastfed, parity among 0 to 3 months breastfed, parity among > = 4 months breastfed, alcohol use, smoking history, hormone replacement therapy (postmenopausal only), oral contraceptive use, BMI, BMI among premenopausal, BMI among postmenopausal
Lee, 2011 [50]	Case-case	1998–2003	1,167 cases	OC use, parity	TN BC	OR (95% CI)	Menarche, oral contraceptive use, parity, limited to women who had a full-term pregnancy, limited to premenopausal women
Lee, 2014 [51]	Cohort study	1993–2009	23,854 cases	Parity (yes vs no)	HER ± BC	OR (95% CI)	Age, breast operation, right/left, multiple tumors, Presence of LVI, NG, pT stage, pN stage, Stage, ER, PR, HER2
Lee, 2020 [52]	Case–control	2011–2018	2,169 cases	Age at menarche, age at first birth, menopausal status, Breastfeeding frequency, parity	Luminal A BC, Luminal B BC, HER2-overexpressing BC, TN BC	OR (95% CI)	Age at menarche, age at first birth, menopausal status, breastfeeding frequency, parity, BMI
Li, 2013 [53]	Case–control	2004- 2010	1,025 cases (941 controls)	Age at menarche, pregnancy, number of live births, age at first live birth, Interval age at menarche-age at FLB, Age at most recent live birth, Time since last birth yrs, breastfeeding	HER2-overexpressing BC, TN BC	OR (95% CI)	Age at menarche, parity, number of live births, age at first live birth, interval between age at menarche and age at first live birth (parous women only), age at most recent live birth, years (parous women only), time since last live birth (years), parous women only), breastfeeding
Li, 2017 [9]	Case–control	2002 to 2010	1,256 cases (1,416 controls)	Menopausal status, Age at menarche, Age at first live birth, Breastfeeding, Age at first birth, Age at postmenopausal	Luminal BC, HER2-enriched BC, TN BC	OR (95% CI)	Age, menopausal status, age at menarche, age at first live birth, breastfeeding, age at post-menopause,
Lorona, 2019 [54]	Case-case	2004–2015	1,701 cases	OC use	Luminal B BC, HER2-overexpressing BC, TN BC	OR (95% CI)	OC use
Ly M, 2012 [55]	Cohort	2008–2011	114 cases	Age of menarche, yrs, Number of full-term pregnancies, Breastfeeding duration, mos, OC use	RH + HER- BC, RH + HER + BC, TN BC	Mean (range)	Age of menarche, yrs, Number of full-term pregnancies, Breastfeeding duration, mos, OC use
Ma, 2010 [56]	Case–control	1994–1998	1,197 cases (2,015 controls)	OC use, Duration of OC use, Age at first use, Duration: menarche and first OC use yrs, Time since last OC use, No. of FTP, Age at FFTP, Duration of breastfeeding mos	Luminal A BC, Luminal B BC, HER2-overexpressing BC, TN BC	OR (95% CI)	OC use, Duration of OC use, Age at first use, Duration: menarche and first OC use yrs, Time since last OC use, No. of FTP, Age at FFTP, Duration of breastfeeding mos
Ma, 2017 [57]	Case–control	1994–2003	2,658 cases (2,448 controls)	Age at menarches, Number of completed pregnancies, Only non-completed pregnancy vs. Never, Age at first completed pregnancy, Duration of breastfeeding mos,	Luminal A BC, Luminal B BC, HER2-overexpressing BC, TN BC	OR (95% CI)	Age at menarche, number of completed pregnancies, only non-completed pregnancy, age at first completed pregnancy, duration of breastfeeding
Mane, 2015 [58]	Cohort	2007–2012	521 cases	Menopausal status	Luminal B BC, HER2-enriched BC, TN BC	OR (95% CI)	Menopausal status, type of surgery, type of chemotherapy, tumor size, outcome (OS)
Martinez, 2013 [59]	Case-case	2007–2011	1,041 cases	Age at menarche, parity, Age at first full-term pregnancy, Time since last full-term pregnancy, Lifetime duration of breastfeeding, Breastfeeding duration per birth, Time from menarche to first pregnancy, Age at menopause, HRT, Hormone contraceptive use	HER2 + BC, TN BC	OR (95% CI)	Age at menarche, parity, age at first full-term pregnancy, time since last full-term pregnancy, lifetime duration of breastfeeding, breast feeding duration per birth, time from menarche to first pregnancy, age at menopause, HRT, Hormone contraceptive use
Millikan, 2008 [60]	Case–control	1993–2001	1,424 cases (2,022 controls)	Menopausal status, Age at menarche, Parity, Age at first full-term pregnancy, Lifetime duration lactation, OC use, HRT (postmenopausal women only), Number of children breastfed, Ave. number months breastfeeding per child, Lactation suppressant use, Parity and lactation, Parity and AFFTP,	HER2 + /ER- BC, Basal-like BC, Luminal A BC, Luminal B BC	OR (95% CI)	Age, race, menopausal status, family history, age at menarche, parity, age at first full-term pregnancy, lifetime duration lactation, alcohol use, smoking duration, oral contraceptive use, hormonal replacement therapy (postmenopausal women only), BMI, WHR
Nichols HB, 2005 [61]	Case–control	1993–1999	682 (649 controls)	Age at menarche, Parity, Age at FFTP, Cumulative lactation mos,	ER + BC, ER- BC, HER2/neu2 + BC, HER2/neu2- BC	OR (95%CI)	Age at menarche, Parity, Age at FFTP, Cumulative lactation mos, BMI
Palmer, 2014 [62]	Case–control	1993–2013	3,698 cases (14,180 controls)	Parity, number of births	TN BC	OR (95% CI)	Nulliparous, parous, number of births (among parous women only), Lactation, Lactation (number of births)
Park, 2016 [63]	Case–control	2001–2007	2,474 cases (3,163 controls)	Age at menarche, Age at menopause among postmenopausal women, Age at first birth among parous women, Period of breastfeeding among breast-fed women, OC use, Breast feeding duration, reproductive factors	Luminal A BC, Luminal B-HER2 Positive BC, HER2-overexpressing BC, TN BC	OR (95% CI)	Family history of breast cancer, reproductive factors (age at menarche, age at menopause among postmenopausal women, age at first birth among parous women, period of breastfeeding among breast fed women, OC) Lifestyle factors (BMI among postmenopausal women, exercise, alcohol drinking)
Park, 2019 [64]	Cohort	1996–2015	158,189 cases	Parity	Luminal A BC, Luminal B (high Ki67), Luminal B (HER2 +), HER2, TN BC	HR (95% CI)	Parity
Phipps, 2008 [65]	Case–control	1997–2004	1,233 cases (1,476 controls)	Age of menarche, parity, No. of live births, Age at 1st live birth, Breastfeeding history, Age at menopause, Hormone therapy use	Luminal BC, HER2-overexpressing BC, TN BC	OR (95% CI)	Age at menarche, parity, no. of live births, age at 1st birth, breastfeeding history, type of menopause, age at menopause, Hormone therapy use
Phipps, 2011 [66, 67]	Case–control	1993–1998	3,116 cases (150,529 controls)	Age at menarche, Age at menopause, Parity, Lifetime duration of use of OC, Age at first use of OC	TN BC	HR (95% CI)	Age at menarche, age at menopause, parity, parous women
Phipps, 2011 [66, 67]	Case–control	1999–2008	10,896 cases (732,727 controls)	Parity, Age at first birth	ER-/PR-/HER2 + BC, TN BC	HR (95% CI)	Parity, Age at 1st birth
Pilewskie, 2012 [68]	Case–control	1998–2011	175 parous, 114 nulliparous women	Parity, Excluding pregnant at diagnosis, Diagnosed when pregnant, Excluding lactating at diagnosis, Diagnosed when lactating, Excluding lactating within 6 months of diagnosis, Diagnosed within 6 months of lactating, Excluding diagnosed during or within 6 months of pregnancy, or while lactating, Diagnosed during or within 6 months of pregnancy, or while lactating	HER2 – BC, TN BC	OR (95% CI)	Time between pregnancy and BC diagnosis, Excluding pregnant at diagnosis, Diagnosed when pregnant, Excluding lactating at diagnosis, Diagnosed when lactating, Excluding lactating within 6 months of diagnosis, Diagnosed within 6 months of lactating, Excluding diagnosed during or wothin 6 months of pregnancy, or while lactating, Diagnosed during or within 6 months of pregnancy, or while lactating
Pizzato, 2020 [69]	Case-case	2008–2014	1,321 cases	Age at menarche, parity	Luminal BH- BC, Luminal BH + BC, HER2 + BC, TN BC	OR (95% CI)	Family history, Density BI-RADS, Education, Age at menarche, Parity
Rauh C, 2015 [70]	Cohort	1995–2008	2,587 cases	Age at menarche, Patients with completed FTP, Patients who had ever used HT, not a previous HT user	Luminal A BC, Luminal B BC, HER2 + BC, TN BC	N (%) mean + -SD	Age at menarche, Patients with completed FTP, Patients who had ever used HT, not a previous HT user
Redondo, 2012 [71]	Case-case	1997–2010	510 cases	Age at first full-term pregnancy, Age at menarche, Age at menopause, Menopausal status, Lifetime duration of breastfeeding, Parity, Parity < = 2, Parity < = 3	TN BC	OR (95% CI)	Age at first full term pregnancy, age at menarche, age at diagnosis, age at menopause, menopausal status, family history, lifetime duration of breastfeeding, parity
Rojas-Lima, 2020 [72]	Case–control	2007–2011	1,045 cases (1,030 controls)	Age at menarche, Age at first pregnancy, Number of pregnancies, Breastfeeding (months), Use of hormonal contraceptive methods, Age at menopause	HER2 + BC, HR + /HER2- BC, HER2 + /HR + BC, TN BC,	OR (95% CI)	Age at menarche, age at first pregnancy, number of pregnancies, breastfeeding (months), use of hormonal contraceptives, age at menopause, estrogenic index
Salagame U.G, 2018 [73]	Case–control	N/A	410 (324 controls)	Current MHT use vs. never	ER + , ER + PR + , Luminal A BC, Luminal B BC	aOR (95%CI)	Current MHT use vs. never
Sanderson, 2021 [74]	Case-case	2012–2018	2,188 cases	HT, age at menarche, OC, parity, age at first birth, breastfeeding, age at menopause	ER + /HER2- BC, HER2 + BC, TN BC	OR (95% CI)	HT, age at menarche, OC, parity, age at first birth, breastfeeding, age at menopause
Saxena, 2010 [19]	Cohort	1995–2006	2,857 invasive BC cases (54,010 controls)	Duration of HT use	ER + or PR + w/HER2 BC-, TN BC, ER + PR + BC, ER-PR- BC	OR (95% CI)	Duration of HT use
Shinde, 2010 [75]	Case-case	2001–2006	2,511 cases	Premenopausal status, HRT duration, HRT duration in postmenopausal women, HRT use, Breastfeeding duration per child, month, Age at menarche, Age at first full-term pregnancy, parity	TN BC	OR (95% CI)	Breastfeeding duration per child, ethnicity, age at diagnosis, age at menarche, age at first full-term pregnancy, parity, premenopausal, HRT duration, HRT use, family history
Sisti, 2016 [17]	Cohort	1976–2006	3,768 cases	Age of menarche, Parity (ever/never), Parity (categorical), Age at first birth, Breastfeeding, Age at menopause, Hormone therapy (HT) use, Time between menarche and first birth (22 yr increase), Duration of pre-menopause (1 yr increase), Duration of menopause (1 yr increase),	Luminal A BC, Luminal B BC, HER2-enrirched BC, Basal-like BC	HR (95% CI)	Age at menarche, parity (ever/never), parity (categorical), age at first birth, breastfeeding, age at menopause, hormone therapy use, Time between menarche and first birth, birth index, duration of pre-menopause, duration of menopause
Song, 2014 [76]	Case-case	2001–2007	3,058 cases	Age at menarche, Age at first pregnancy, Number of pregnancies, Number of children, Age at first full-term pregnancy among parous women, Breastfeeding among parous women, Duration of endogenous estrogen exposure before the first full-term pregnancy, years, Duration of total endogenous estrogen exposure years	HR- HER2- BC, HR + HER2 +	OR (95% CI)	Age, family history of breast cancer, BMI, Age at menarche, number of children, age at first full-term pregnancy among parous women, breastfeeding among parous women, duration of endogenous estrogen exposure before first full-term pregnancy, duration of total endogenous estrogen exposure
Sung, 2016 [77]	Case–control	N/A	5,040 cases	Age at menarche, Parity, Age at first full-term pregnancy, Number of live births, Breastfeeding	Luminal A Basal BC	OR (95% CI)	Family history of breast cancer, age at menarche, parity, age at first full-term pregnancy, number of live births, breastfeeding, BMI
Sung, 2020 [78]	Case-case	2003–2015	2,977 cases	Parity, breastfeeding	HR Negative	OR (95% CI)	BMI, Parity/age at first birth, Breastfeeding
Tamimi, 2012 [18]	Case-case	1976–1996	2,022 cases	Age at menarche, Parity, Age at first birth, Age at menopause, Menopausal status/PMH use, Lactation	Luminal A BC, Luminal B BC, HER2 BC, Basal-like BC	RR (95% CI)	Age at menarche, BMI at age 18, Weight gain since 18, Parity, age at first birth, age at menopause, menopausal status/PMH use, alcohol consumption, lactation, family history of breast cancer
Trivers, 2009 [79]	Case–control	1990 and 1992	476 cases (913 controls)	Age at first birth, yrs, No. of FTB, Recency of birth yrs, Breastfeeding, breastfeeding mos, Age at menarche, yrs	Luminal A BC, Luminal B BC, HER2-overexpressing BC, TN BC	OR (95% CI)	Race, age at diagnosis, education, poverty index, smoking status, alcohol, age at menarche, age at first birth, no of full-term births, recency of birth, breastfeeding, breastfeeding months, BMI, Physical activity-year before interview
Tsakountakis, 2005 [80]	Case–control	1996–2002	384 cases (566 controls)	Age at FFP, Abortion/miscarriage, OC use, Age at menarche, Use of estrogen	HER2/neu + BC, HER2/neu—BC	OR (95% CI)	Age at first full pregnancy, body mass index, abortion or miscarriage, first degree family history, use of oral contraceptives, age of menarche
Turkoz, 2013 [81]	Case-case	1983–2011	1,884 cases	Age at menarche, Age at menopause, Age at first full-term pregnancy, Number of children, Breastfeeding, OC use, HRT use, In vitro fertilization	Luminal A BC, Luminal B BC, HER2-overexpressing BC, TN BC	OR (95% CI)	Age, family history, blood type, use of hand, age at menarche, age at menopause, age at first full term pregnancy, number of children, breastfeeding, oral contraceptive use, HRT use, In vitro fertilization, obesity, smoking
Von Au, 2017 [82]	Case-case	2009–2014	1,082 cases	Number of children, Duration of breastfeeding, Age at first birth	Non-luminal BC vs. luminal like BC	OR (95% CI)	Number of children, duration of breastfeeding, age at diagnosis, BMI, Smoking behavior, age at first birth
Wang, 2020 [83]	Case–control	2012–2017	3,792 cases (4,182 controls)	Parity vs no parity, Number of live births, Age at first live birth, Breastfeeding (parous women),	Luminal A BC, Luminal B BC, HER2-enriched BC, TN BC	OR (95% CI)	Age of menarche, parity vs. no parity, number of live births, age at first live birth, breastfeeding (parous women)
Work M.E., 2014 [84]	Case-case	1995–2004	4,011 cases (2,997 controls)	High parity and breastfeeding, OC use commenced prior 1975, OC use, Parity, Breastfeeding duration, Parous, never breastfed vs. Nulliparous, Parous, ever breastfed vs. nulliparous	ER-PR- BC, HR + BC	OR (95% CI)	High parity and breastfeeding, OC use commenced prior 1975, OC use, Parity, Breastfeeding duration, Parous, never breastfed vs. Nulliparous, Parous, ever breastfed vs. nulliparous
Xing, 2010 [85]	Case–control	2001–2009	1,417 cases (1,587 controls)	Age of menarche, Parity, Breastfeeding, Age at first live birth, Menopausal status, Age at menopause, Spontaneous abortion, Induced abortion	Luminal A BC, Luminal B BC, HER2-overexpressing BC, TN BC	OR (95% CI)	Age at menarche, parity, breastfeeding, age at first live birth, menopausal status, age at menopause, first-degree family history of breast cancer, hysteromyoma history, spontaneous abortion, induced abortion
Yang, 2007 [86]	Case–control	2000–2003	842 cases (2,502 controls)	Age at menarche (per 2 yr increase), Number of full-term births, Age at first full-term birth (per 5 yr increase), Age at menopause (per 5 yr increase)	Luminal A BC, Luminal B BC, HER2-overexpressing BC, Basal-like BC	OR (95% CI)	Age at menarche, number of fullterm births, age at first full term birth, age at menopause, BMI in premenopausal, BMI in postmenopausal, Family history
Ye, 2019 [87]	Case–control and Case-case	2014–2017	1,118 cases (2,284 controls)	History of abortion, History of spontaneous abortion, history of induced abortion, Menopausal status, Age at menopause	Luminal A BC, Luminal B BC, HER2-overexpressing BC, TN BC	OR (95% CI)	Menopausal status, age at menopause, history of abortion, history of spontaneous abortion, breast density, tumor location
Yuan X., 2009 [88]	Cohort	2004–2009	274 cases	Age at menarche yrs, Age at first live birth yrs, Age at menopause, Ever breastfeeding	HER2 + BC, HER2—BC	N(%) Mean ± SD	Age at menarche yrs, Age at first live birth yrs, Age at menopause, Ever breastfeeding
Zhang, 2019 [89]	Cohort	2004–2014	8,067 cases	Age of menarche, Number of pregnancy, Age at first full term pregnancy (years), Number of live births, Months of breastfeeding, Abortion, OC, HRT, menopause	Luminal B BC, HER2-enriched BC, Basal-like BC	OR (95% CI)	BMI, Age of menarche, number of pregnancy, age at first full term pregnancy, number of live births, months of breastfeeding, abortion, oral contraceptive, HRT, Menopause, Benign breast disease, smoking, alcohol drinking, negative events, first degree family history of breast cancer, breast density

### Age at menarche

Forty-six studies evaluated the association between age at menarche and BC subtypes (twenty-four case–control studies [9, 22, 28, 36, 37, 39–42, 44, 46, 48, 53, 57, 61, 63, 65, 66, 72, 79, 80, 83, 85, 86], fourteen case-only studies [20, 27, 30, 35, 43, 59, 69, 71, 74–77, 81, 89], three case–control/case-case studies [24, 52, 60], and five cohort studies [17, 18, 55, 70, 88]), and were included in the systematic review. Among the cohort and case–control studies, later age at menarche was associated with lower risk of BC in the majority of studies regardless of subtype [9, 17, 18, 22, 24, 28, 36, 37, 39–42, 44, 46, 48, 52, 53, 55, 57, 60, 61, 63, 65, 66, 70, 72, 79, 80, 83, 85, 86, 88], of which fourteen studies [17, 18, 24, 39, 40, 42, 52, 57, 63, 72, 79, 83, 85, 86] were luminal A, nine studies [17, 24, 39, 42, 52, 57, 63, 85, 86] were luminal B, twelve studies [17, 22, 24, 39, 42, 44, 53, 63, 65, 79, 83, 85] were HER2, and eighteen studies were TNBC [9, 18, 22, 37, 39, 40, 46, 52, 53, 57, 60, 63, 65, 66, 72, 79, 85, 86]. Among the case-only studies [20, 24, 27, 30, 35, 43, 52, 59, 60, 69, 71, 74–77, 81, 89], compared to luminal A, later age of menarche was associated with lower risk of luminal B in seven studies [20, 24, 30, 43, 52, 69, 89], HER2 subtype in six studies [20, 30, 59, 74, 76, 89], and TNBC in eight studies [27, 30, 52, 60, 71, 74, 76, 89]. Sung et.al. compared luminal A basal-positive with luminal A basal-negative cases but reported a non-significant lower association between age at menarche and BC subtypes [77]. Yaun et. al., reported younger age at menarche was more often observed in patients with HER2-positive compared to patients with HER2- negative status, while Ly et. al., and Rauh et. al., observed no significant differences between mean age of menarche and BC subtypes [55, 70, 88].

**Fig. 2:**
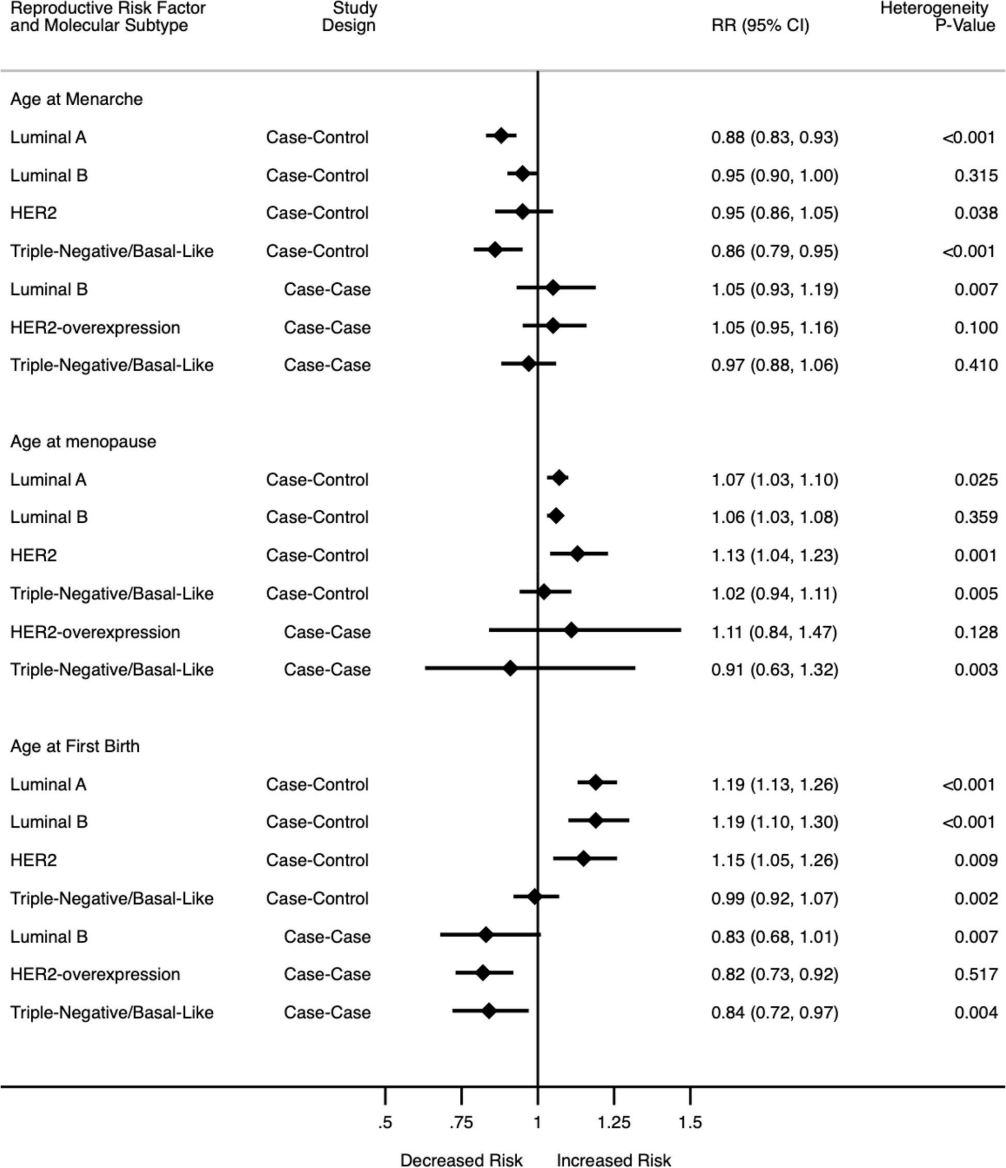
Summary of meta-analysis results for age at menarche, age at menopause and age at first birth (Estimates for case–control studies also include cohort studies)

In meta-analysis of two cohort and twenty-seven case–control studies (Fig. 2; Supplementary Fig. 1), later vs earlier age at menarche was associated with a 12% lower risk of luminal A (RR: 0.88, 95% CI: 0.83, 0.93, *I*^2^ = 64.1%, *p* < 0.001) and 14% lower risk of TNBC (RR:0.86, 95% CI: 0.79, 0.95, *I*^2^ = 57.6%, *p* < 0.001). Associations with luminal B (RR: 0.95, 95% CI: 0.90, 1.00, *I*^2^ = 12.1%, *p* = 0.315) and HER2-overexpressing (RR: 0.95, 95% CI: 0.86, 1.05, *I*^2^ = 37.2%, *p* = 0.038) subtypes were lower but not significant. There was evidence of heterogeneity and publication bias among the studies (Egger test: -0.631, *p* = 0.004). In analysis of thirteen case-case studies (Fig. 2; Supplementary Fig. 2), compared to luminal A, other subtypes were not significantly associated with age at menarche. There was some heterogeneity among the case-case studies, but no evidence of publication bias (Egger test:0.43, *p* = 0.309).

### Age at menopause

Twenty-one studies evaluated the association between age at menopause and BC subtypes (ten case–control studies, seven case-case studies, one case–control/case-case study, and three cohort studies). In the cohort studies and eleven case–control studies [9, 17, 18, 36, 38, 40, 46, 48, 52, 53, 63, 65, 66, 72, 85–88], later age at menopause was associated with higher risk among nine luminal A studies [17, 18, 38, 40, 63, 72, 85–87], four luminal B studies [17, 18, 40, 86], ten HER2-overexpressing studies [17, 18, 38, 40, 46, 63, 65, 72, 86, 87], and eleven TNBC/basal-like studies [17, 18, 36, 38, 46, 63, 65, 66, 72, 85, 86]. Ye et al. [87] and Yuan et al. [88] observed no significant differences in mean age at menopause and BC subtypes. Among the eight case-case studies [35, 59, 69, 74, 76, 77, 87, 89], compared to luminal A, later age of menopause was associated with higher risk of HER2 subtype in three studies [35, 74, 87], but conflicting associations with risk of TNBC in four studies [35, 71, 76, 81].

In meta-analysis of two cohort and eleven case–control studies (Fig. 2; Supplementary Fig. 3), later vs. earlier age at menopause was associated with a 7% higher risk of luminal A (RR:1.07, 95% CI: 1.03, 1.10, *I*^2^ = 56.1%, *p* = 0.025), 6% higher risk of luminal B (RR:1.06, 95% CI: 1.03, 1.08, *I*^2^ = 9.2%, *p* = 0.359) and 13% higher risk of HER2-overexpressing (RR:1.13, 95% CI: 1.04, 1.23, *I*^2^ = 69.6%, *p* = 0.001), while the association with TNBC subtype (RR:1.02, 95% CI: 0.94, 1.11, *I*^2^ = 58.8% *p* = 0.005 was not significant. There was no evidence of publication bias (Egger’s test: 0.615, *p* = 0.074). In the case-case analyses (Fig. 2; Supplementary Fig. 4), later age at menarche was not significantly associated with any of the subtypes compared to luminal A. There was some heterogeneity in the studies, but no evidence of publication bias (Egger’s test -1.04, *p* = 0.06).

### Age at first birth

Forty-three studies evaluated the association between age at first birth and BC subtypes (twenty-three case–control studies, fourteen case-case studies, three case–control/case-case studies, and three cohort studies). In the cohort and case–control analysis [9, 17, 18, 22, 24, 28, 37–40, 42, 44, 46, 52, 53, 56, 57, 61, 65, 67, 72, 79, 80, 83, 85, 88], later age at first birth/nulliparity vs. earlier age /parity was associated with higher risk in fifteen studies [9, 17, 18, 24, 38–40, 42, 52, 56, 57, 60, 63, 79, 86] except one [85] evaluating luminal A, ten [17, 24, 38–40, 42, 52, 57, 79, 86] studies evaluating luminal B, thirteen [17, 18, 24, 28, 38, 39, 42, 53, 61, 67, 72, 80, 85] studies evaluating HER2 and twelve [17, 18, 37–40, 42, 52, 56, 67, 72, 79] studies evaluating TNBC. Yuan et al. did not find any difference in the mean age at first birth comparing HER2-positive vs. HER2-negative [88]. In case-case analysis [20, 24, 27, 30, 35, 43, 49, 52, 59, 60, 71, 74–77, 81, 82, 89], compared to luminal A, later age/nulliparity was associated with lower risk of luminal B in five [24, 43, 52, 81, 89] studies, three studies [24, 49, 60] evaluating HER2-overexpression, and six TNBC studies [27, 43, 49, 71, 74, 89]. Five studies had other comparison groups other than luminal A [43, 75, 77, 81, 82], and found later age at first birth was associated with a higher risk of luminal B, lower risk of HER2 and a mixed association with TNBC.

In the meta-analysis of two cohort and twenty-six case–control studies (Fig. 2; Supplementary Fig. 7), later age at first birth/nulliparity vs. younger age was associated with a 19% higher risk of luminal A (RR:1.19 95% CI: 1.13, 1.26, *I*^2^ = 87.6%, *p* = 0.000), 19% for luminal B (RR:1.19, 95% CI: 1.10, 1.30, *I*^2^ = 76.6%, *p* < 0.001) and 15% for HER2 (RR:1.15, 95% CI: 1.05, 1.26, *I*^2^ = 45.2%, *p* = 0.009), while there was no difference in risk for TNBC (RR:0.99, 95% CI: 0.92, 1.07, *I*^*2*^ = 51.3%, *p* = 0.002). There was heterogeneity among the studies and evidence of publication bias (Egger 0.778, *p* = 0.001). In meta-analysis of case-case studies (Fig. 2; Supplementary Fig. 8), compared to luminal A, later age at first birth/nulliparity vs. younger age was associated with a 18% lower risk of HER2 subtype (RR: 0.82, 95% CI: 0.73, 0.92, *I*^2^ = 0.0%, *p* = 0.517) and 16% lower risk of TNBC (RR: 0.84, 95% CI: 0.72, 0.97, *I*^2^ = 55.6%, *p* = 0.004). Lower risk was also observed in studies evaluating luminal B but the association was not significant (RR: 0.83, 95% CI: 0.68, 1.01, *I*^*2*^ = 60.6%, *p* = 0.007). There was heterogeneity among the studies but no evidence of publication bias (Egger’s test -1.043, *p* = 0.059).

### Menopausal status

Eighteen studies evaluated the association between menopausal status and BC subtypes (five case–control studies, seven case-case studies, three case–control/case-case studies, and three cohort studies). In the cohort and case–control study analysis [9, 18, 22, 29, 38, 45, 52, 60, 65, 85, 87], post/peri vs pre/natural menopause was associated with a lower risk of luminal A in four studies [18, 38, 52, 85], luminal B in four studies [18, 38, 52, 85], and TNBC in seven studies [9, 18, 38, 52, 65, 85, 87], but higher risk in five HER-2 overexpressing studies [22, 38, 45, 52, 65]. Ihemelandu et al. observed that molecular subtypes did not differ by menopausal status, however basal cell-like subtype also showed an age-specific bimodal distribution with a peak in the < 35 and 51 to 65 years age groups [45]. Chauhan et. al., reported a higher percentage of Her2-neu receptor in post-menopausal compared to pre-menopausal women, while the opposite was seen in TNBC patients [29]. In the ten case-case analysis studies [35, 49, 52, 58, 60, 71, 75, 76, 87, 89], compared to luminal A, post/perimenopausal status was associated with a higher risk in four [52, 58, 60, 89] luminal B studies, six HER2-overexpressing studies [49, 52, 58, 60, 87, 89], and six TNBC studies [49, 52, 58, 60, 75, 89].

**Fig. 3:**
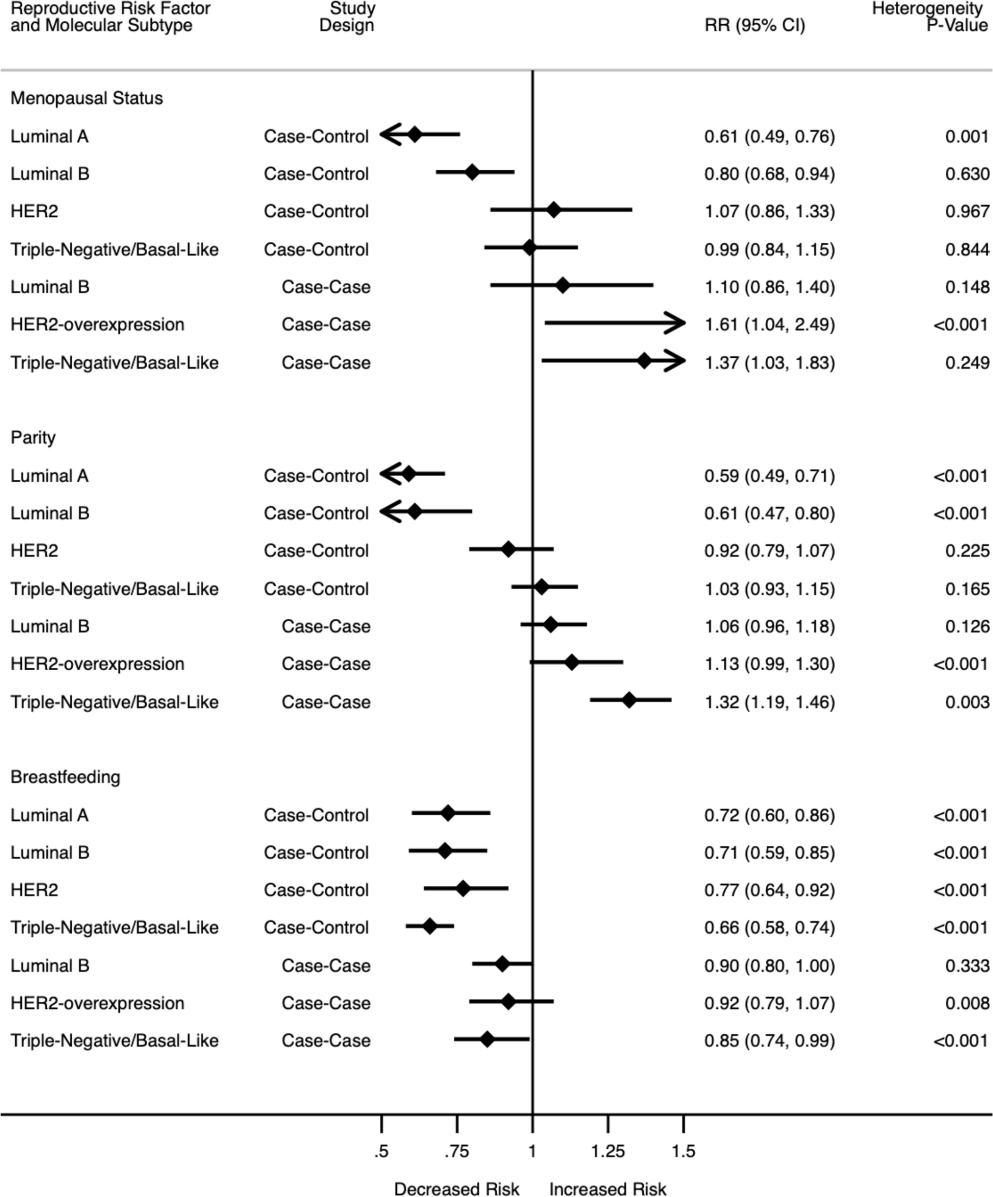
Summary of meta-analysis results for menopausal status, parity, and breastfeeding (Estimates for case–control studies also include cohort studies)

In the meta-analysis of one cohort and six case–control studies (Fig. 3; Supplementary Fig. 5) post/peri menopausal status was associated with a 39% lower risk of luminal A (RR: 0.61, 95% CI: 0.49, 0.76, *I*^2^ = 79.0%, p = 0.001), 20% lower risk of luminal B (RR:0.80, 95% CI: 0.68, 0.94, *I*^2^ = 0%, p = 0.630); however the association was statistically non-significantly higher for HER2-overexpressing (RR: 1.07, 95% CI: 0.86, 1.33, *I*^2^ = 0.0%, *p* = 0.967) and lower for TNBC (RR: 0.99, 95% CI: 0.84, 1.15, *I*^*2*^ = 0%, *p* = 0.844). There was heterogeneity among luminal A studies, as well as evidence of publication bias (Egger’s test 2.17, *p* < 0.001). In the case-case meta-analysis (Fig. 3; Supplementary Fig. 6), compared to luminal A, post/peri-menopausal status was associated with 61% higher risk of HER2-overexpressing (RR: 1.61 95% CI: 1.04, 2.49, *I*^*2*^ = 78.8%, *p* < 0.001), though the association was not significant with luminal B (RR: 1.10, 95% CI: 0.86, 1.40, *I*^*2*^ = 41.0%, *p* < 0.001) or TNBC (RR: 1.07, 95% CI: 0.74, 1.56, *I*^*2*^ = 60.5%, *p* = 0.019). There was significant heterogeneity but no evidence of publication bias (Egger -0.138, *p* = 0.910).

### Parity

Fifty-three studies evaluated the association between parity and BC subtypes (twenty-three case–control studies, nineteen case-case studies, three case–control/case-case studies, and eight cohort studies). In the cohort and case–control studies [16–18, 21, 22, 24, 26, 31, 34, 39, 40, 42, 46–48, 51–53, 55–57, 60–62, 64–67, 72, 79, 80, 83, 85, 86], higher parity was associated with a lower risk in fifteen studies [16–18, 26, 39, 40, 42, 47, 52, 56, 57, 60, 79, 83, 86] evaluating luminal A, twelve [16, 17, 39, 40, 42, 47, 52, 56, 57, 79, 83, 86] studies evaluating luminal B, twelve [18, 26, 39, 42, 53, 56, 57, 61, 65, 67, 79, 83] studies evaluating HER2-overexpression, and higher risk in eleven [22, 26, 37, 42, 46, 47, 53, 57, 65, 79, 83] studies evaluating TNBC. In 22 case-case studies [20, 24, 27, 30, 35, 43, 49, 50, 52, 59, 60, 69, 71, 74–78, 81, 82, 84, 89] compared with luminal A, higher parity was associated with higher risk in five luminal B studies, [24, 27, 52, 69, 78] seven HER2-overexpressing studies, [24, 43, 59, 69, 74, 78, 81], and six TNBC studies [49, 69, 75, 76, 78, 81]. Ten studies had either different comparison groups other than luminal A or reported proportions to evaluate associations [31, 34, 43, 50, 51, 55, 75, 77, 81, 82]. These studies found a lower risk of HER2 and mixed associations between higher parity and luminal B and TNBC.

In the meta-analysis of three cohort and twenty-six case–control studies (Fig. 3; Supplementary Fig. 9), higher parity was associated with a 39% lower risk of luminal A (RR: 0.59, 95% CI: 0. 49, 0.71, *I*^2^ = 92.4%, *p* < 0.001) and 39% lower risk of luminal B (RR: 0.61, 95% CI: 0.47, 0.80, *I*^2^ = 91.9%, *p* < 0.001). There was no significant association for HER2-overexpressing BC (RR: 0.92, 95% CI: 0.79, 1.07, *I*^2^ = 17.7%, *p* = 0.225) or TNBC (RR: 1.03, 95% CI: 0.93, 1.15, *I*^2^ = 21.0%, *p* = 0.165). There was no heterogeneity between the studies and no evidence of publication bias (Egger’s test 0. 465, *p* = 0.304). For the sixteen case-case studies (Fig. 3; Supplementary Fig. 10), compared to luminal A, higher parity was associated with a 32% higher risk of TNBC (RR: 1.32, 95% CI: 1.19, 1.46, *I*^2^ = 45.7%, *p* = 0.003). No significant association was found in luminal B (RR: 1.06, 95% CI: 0.96, 1.18) and HER2-overexpressing BC (RR: 1.13, 95% CI: 0.99, 1.30). There was heterogeneity between the groups and evidence of publication bias (Egger’s test: 1.07, *p* = 0.007).

### Breastfeeding

Forty-seven studies evaluated the association between breastfeeding and BC subtypes (twenty-five case–control studies, fifteen case-case studies, three case–control/case-case studies, and four cohort studies). In the cohort and case–control studies [9, 16–18, 21, 22, 24, 28, 36–39, 41, 42, 44, 46–48, 52, 53, 56, 57, 60–63, 65, 72, 79, 83, 88], breastfeeding was associated with lower risk in all subtypes: thirteen studies [17, 18, 39, 42, 47, 56, 57, 60, 63, 72, 79, 83, 85] evaluating luminal A, thirteen studies [17, 18, 24, 39, 42, 47, 56, 57, 63, 72, 79, 83, 85] evaluating luminal B, 15 studies [9, 18, 24, 28, 39, 42, 44, 47, 52, 61, 63, 72, 79, 83, 85] evaluating HER2-overexpression, and twenty-three studies evaluating TNBC [9, 17, 18, 21, 22, 24, 39, 42, 46–48, 52, 53, 56, 57, 60, 62, 63, 65, 72, 79, 83, 85]. In the case-case analysis [24, 30, 35, 43, 49, 52, 59, 60, 71, 74–78, 81, 82, 84, 89], ever/higher duration of breastfeeding was associated with lower risk in all subtypes: five [30, 52, 60, 84, 89] luminal B studies, ten HER2-overexpressing studies [24, 43, 49, 52, 59, 60, 74, 76, 81, 84], and 13 evaluating TNBC [24, 30, 35, 49, 52, 60, 71, 74–76, 78, 81, 84] vs. luminal A. Six studies had different comparison groups or used proportions [43, 75, 77, 81, 82, 88], and found a higher association between longer duration of breastfeeding with HER2 subtype and mixed associations between longer duration of breastfeeding and luminal B and TNBC.

In the meta-analysis of thirty studies (Fig. 3; Supplementary Fig. 11), ever/longer duration of breastfeeding was associated with lower risk of all subtypes compared to the controls; 28% lower risk of luminal A (RR: 0.72, 95% CI: 0.60, 0.86, *I*^2^ = 93.6%, *p* < 0.001), 29% lower risk of luminal B (RR: 0.71, 95% CI: 0.59, 0.85, *I*^2^ = 82.5%, *p* < 0.001), 23% lower risk of HER2-overexpressing (RR: 0.77, 95% CI: 0.64, 0.92, *I*^2^ = 74.3%, *p* < 0.001), and 42% lower risk of TNBC (RR: 0.66, 95% CI: 0.58, 0.74, *I*^2^ = 58.8%, p < 0.001) with significant heterogeneity between the studies, but no evidence of publication bias (Egger: -0.333, *p* = 0.499). In case-case studies (Fig. 3; Supplementary Fig. 12) compared to luminal A, there was 15% lower risk of TNBC (RR: 0.85, 95% CI: 0.74, 0.99, *I*^2^ = 58.8%, *p* < 0.001), while luminal B breast cancer (RR: 0.90 95% CI: 0.80, 1.00) and HER2-overexpressing BC (RR: 0.92 95% CI: 0.79, 1.07) results were not significant. There was heterogeneity between the studies reporting HER2 subtype but no evidence of publication bias (Egger’s test -0.27, *p* = 0.529).

### Oral contraceptive (OC) use

Twenty-three studies evaluated the association between OC use and BC subtypes (thirteen case–control studies, nine case-case studies, and two case–control/ case-case studies). In the case–control analysis [23–25, 28, 37–41, 44, 56, 60, 63, 66, 80], OC use was associated with higher risk in all subtypes: three [24, 38, 63] luminal A studies, six [24, 38–40, 56, 63] luminal B studies, seven [24, 37–39, 44, 56, 63] HER2-overexpressing studies and eight [23–25, 37–39, 63, 66] TNBC studies. In the case-case analysis in comparison to luminal A subtype [24, 49, 50, 54, 59, 60, 74, 76, 81, 84, 89], OC use was associated with a higher risk in one [81] study evaluating luminal B, six [24, 49, 60, 74, 76, 84] studies evaluating HER2 and six [24, 50, 54, 74, 76, 84] studies evaluating TNBC. Two studies had different comparison groups [50, 81]. Turkoz et al. observed no significant difference with OC use (< 2 years, ≥ 2 to 5 years and ≥ 5 years vs. no use) among breast cancer subtypes comparing luminal A to non-luminal A and luminal B to non-luminal B [81]. Lee et al. did not find any risk of TNBC with OC use compared to non-TNBC [50].

**Fig. 4:**
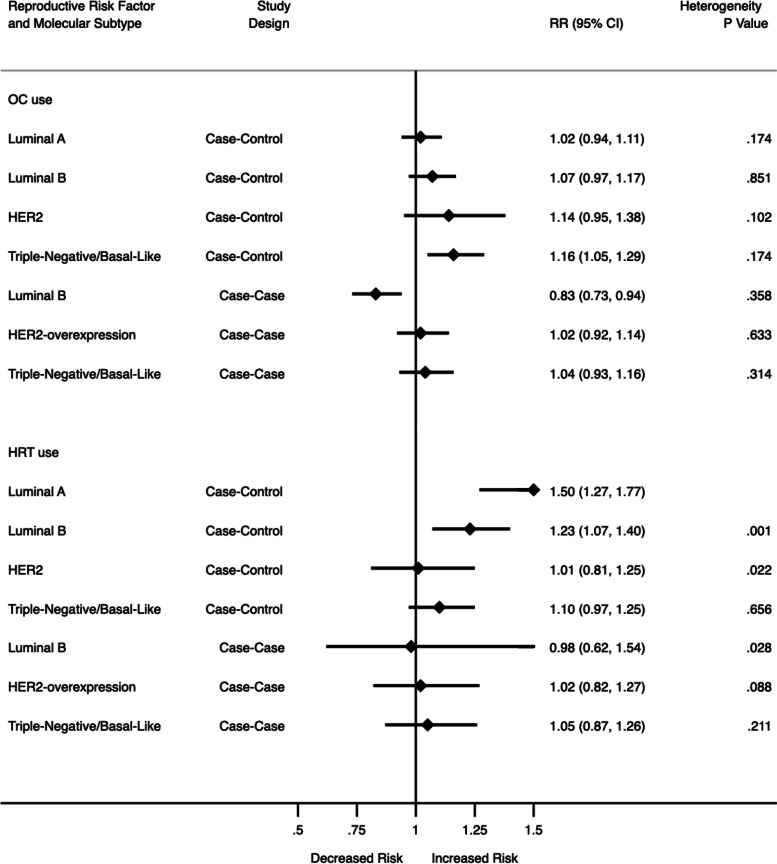
Summary of meta-analysis results for OC use and HRT use (Estimates for case–control studies also include cohort studies)

In the meta-analysis of fifteen case–control studies (Fig. 4; Supplementary Fig. 13), ever/longer duration OC use was associated with a 16% higher risk of TNBC (RR: 1.16, 95% CI: 1.05, 1.29, *I*^2^ = 26.0%, *p* = 0.174). The higher association was not statistically significant for luminal A (RR:1.02, 95% CI: 0.94, 1.11), luminal B (RR: 1.07, 95% CI: 0.97, 1.17) or HER2 (RR: 1.14, 95% CI: 0.95, 1.38). There was no heterogeneity between these studies, but there was publication bias (Egger 0.545, *p* = 0.030). In the meta-analysis of nine case-case studies (Fig. 4; Supplementary Fig. 14) compared to luminal A, ever/longer duration OC use was associated with a 17% lower risk of luminal B (RR: 0.83, 95% CI: 0.73, 0.94, *I*^*2*^ = 9.4%, *p* = 0.358). There was a higher risk of HER2-overexpression (RR: 1.02, 95% CI: 0.92, 1.14) and TNBC subtypes (RR: 1.04, 95% CI: 0.93, 1.16), but these were not significant. There was no evidence of heterogeneity or publication bias (Egger: -0.36, *p* = 0.527).

### Hormone replacement therapy (HRT)

Twenty-one studies evaluated the association between HRT use and BC molecular subtypes (ten case–control studies, six case-case studies, one case–control/case-case study, and four cohort studies). In the cohort and case–control analysis [17, 18, 28, 33, 36, 38, 42, 44, 60, 65, 70, 72, 73, 80], any use of HRT was associated with higher risk in all BC subtypes: all seven luminal A studies [17, 33, 38, 42, 70, 72, 73] and lower risk among subgroups of estrogen and progesterone therapy (≤ 5 years vs. never) [17] and women with BMI < 25 [33], higher risk in three [17, 38, 72] luminal B studies, four [36, 42, 60, 72] HER2-overexpressing studies and six [17, 19, 33, 38, 42, 72] TNBC studies. In the case-case analysis [49, 59, 60, 74, 75, 81, 89], ever use of HRT was associated with a lower risk in three luminal B studies [60, 81, 89], while higher risk was observed in five [59, 60, 74, 81, 89] HER2 studies, and one [74] TNBC study compared to luminal A. Three studies that were excluded from the meta-analysis because they had different comparison groups or reported frequencies [70, 75, 81] found an association between higher duration of HRT use with higher risk of luminal B and HER2, and mixed results with TNBC.

In the meta-analysis of eleven case–control studies and two cohort studies (Fig. 4; Supplementary Fig. 15), ever use of HRT was associated with a 50% higher risk of luminal A (RR: 1.50, 95% CI: 1.27, 1.77, *I*^2^ = 90.3%, *p* < 0.001) and 23% higher risk of luminal B (RR: 1.23, 95% CI: 1.07, 1.40, *I*^2^ = 60.0%, *p* = 0.001), however no significant association with HER2-overexpressing (RR: 1.01, 95% CI: 0.81, 1.25) and TNBC (RR: 1.10, 95% CI: 0.97, 1.25) with significant heterogeneity between the studies, but no publication bias (Egger -0.77, *p* = 0.108). For the five case-case studies (Fig. 4; Supplementary Fig. 16), any use of HRT was not associated with any BC molecular subtypes. There was some heterogeneity but no evidence of publication bias: (Egger: -1.61, *p* = 0.123).

### Pregnancy

Four studies [38, 57, 72, 89] evaluated the association between pregnancy and BC molecular subtypes (three case–control studies and one case-case study). However, we did not have a sufficient number of studies for meta-analysis; studies by Rojas-Lima, Ma, and Ellingford-Dale et al. showed a similar association between the number of pregnancies with lower risk of luminal B, HER2-overexpressing, and TNBC [38, 57, 72]. In Zhang et al., the results show a higher risk of luminal B breast cancer and lower risk of HER2-overexpressing and TNBC with number of pregnancies [89].

### Years since last birth/pregnancy

Six studies [31, 32, 34, 53, 59, 68] evaluated the association between years since last birth/pregnancy and BC molecular subtypes (two case–control studies, one case-case, and two cohort studies). DeMudler et. al., reported that diagnosis of BC within 5 years after last birth was proportionally more likely to be TNBC and HER2-overexpressing BC compared to luminal A breast cancer subtype [34]. Pilewskie et. al., found BCs diagnosed in parous women 0–2 years from last parity were more likely to be diagnosed with TNBC and HR negative compared to nulliparous women [68]. However, Li et. al [53], and Martinez et al. [59] did not find any significant association between years since last birth and any BC molecular subtypes. Martinez et al. adjusted for age at menopause, though the four other studies did not adjust for age or menopausal status (potential confounding variables).

### Abortion

Five studies [37, 44, 80, 85, 87] evaluated the association between abortion and BC molecular subtypes (four case–control studies and one case–control/case-only study), which were included in the systematic review. However, we did not have enough studies to conduct a meta-analysis. Ye et al. reported no significant association with lower risk for all subtypes except TNBC, which showed a non-significant higher risk [87], while Huang et al. reported no significant association with HER2 + subtype but the studies had different reference groups [44]. Dolle et al. reported a non-significant association for higher risk in TNBC [37]. Xing et al. reported a significant association for lower risk of luminal A and luminal B breast cancer, but a lower non-significant association for HER2 + and TNBC for spontaneous abortion [85]. For induced abortion, Xing et al. reported a significant association for higher risk of luminal A, and a higher non-significant association for luminal B, HER2 overexpressing, and TNBC subtypes [85]. Tsakountakis et. al. reported that history of ever abortion was associated with a lower risk of HER-2/neu + tumors among postmenopausal women [80].

### Summary of meta-analyses

Out of eight reproductive factors included in this meta-analysis, three were consistently associated with BC risk across subtypes: age at menopause, age at first birth, and breastfeeding, with the strongest associations observed in the luminal A subtype among case–control and cohort studies (Table 2). The associations for parity, age at menarche, OC use, HRT use, and menopausal status were less consistent, and the strongest associations were observed for the luminal B subtype (Table 2). In the case-case analysis, the strongest associations were menopausal status and age at first birth for HER2-enriched and TNBC subtypes, but not for luminal B breast cancer (Table 3). The associations for parity, age at menarche, OC use, HRT use, and breastfeeding were less consistent and the strongest associations overall were observed in TNBC (Table 3).

**Table 2:** Summary of meta-analysis results for case–control/cohort studies (*n*=42 studies)

Reproductive factors	Contrast	Luminal A/ ER + , PR + , HER-	Luminal B/ ER + , PR + , HER +	HER2-overexpressing/ER-, PR-, HER +	Triple Negative/ basal-like/ER-, PR-, HER-
Age at menarche (*n* = 29)	Later vs. earlier	S-	NO	NO	S-
Age at menopause (*n* = 13)	Later vs. earlier	S +	S +	S +	NS +
Menopausal status (*n* = 7)	Post vs. pre/peri	S-	S-	NS +	NS-
Age at first birth (*n* = 28)	Nulliparous/later vs. earlier age at FB	S +	S +	S +	NS +
Parity (*n* = 29)	Higher parity vs. low/nulliparity	S-	S-	NS-	NS +
Breastfeeding (*n* = 30)	Ever /longer vs. never/shorter	S-	S-	S-	S-
OC use (*n* = 15)	Ever/longer vs. never/shorter	NS +	NS +	NS +	S +
HRT (*n* = 13)	Ever/longer vs. never/shorter	S +	S +	NS +	NS +

*Abbreviations*: *S-* Significant lower risk, *S+* Significant higher risk, *NS+* Non-significant higher risk, *NS-* Non-significant lower risk, *NO* No difference (0.95 < RR < 1.05 and NS), *FB* First birth, *OC* Oral contraceptive

*HRT* Hormone replacement therapy, *HER2* Human epidermal growth factor receptor 2

**Table 3:** Summary of meta-analysis results for case-case analyses (Reference group: Luminal A; *n* = 18 studies)

Reproductive factors	Contrast	Luminal B/ ER + , PR + , HER +	HER2-overexpressing/ER-, PR-, HER +	Triple Negative/ basal-like/ER-, PR-, HER-
Age at menarche (*n* = 13)	Later vs. earlier	NS +	NS +	NS-
Age at menopause (*n* = 6)	Later vs. earlier	–	NS +	NS-
Menopausal status (*n* = 7)	Post vs. pre/peri	NS +	S +	S +
Age at first birth (*n* = 12)	Nulliparous/later vs. earlier age at FB	NS-	S-	S-
Parity (*n* = 16)	Higher parity vs. low/nulliparity	NS +	NS +	S +
Breastfeeding (*n* = 10)	Ever /longer vs. never/shorter	NS-	NS-	S-
OC use (*n* = 9)	Ever/longer vs. never/shorter	S-	NS +	NS +
HRT (*n* = 5)	Ever vs. never	NS-	NS +	NS +

*Abbreviations*: *S-* Significant lower risk, *S* + Significant higher risk, *NS* + Non-significant higher risk, *NS-* Non-significant lower risk, *FB* First birth, *OC* Oral contraceptive, *HRT* Hormone replacement therapy

## Discussion

This systematic review and meta-analysis summarizes the current published literature examining eleven reproductive factors and the risk of breast cancer molecular subtypes. To ensure consistency in contemporary classifications of BC based on molecular subtypes, the scope of this review was limited to luminal A, luminal B, HER2-overexpressing, and TNBC/basal-like breast cancer. In the 75 included studies, we observed that the strength and consistency of associations across subtypes differed by risk factors.

Late age at menarche was protective for all four BC subtypes. However, the other factors were most consistently associated with the luminal A subtype. Except for OC use, most of the associations between reproductive risk factors were observed for the luminal subtypes, similar to a prior systematic review showing stronger associations among hormone receptor-positive BC [3].

The prior meta-analysis on this topic evaluated three risk factors: parity, age at first birth, and breastfeeding [6]. Our meta-analysis updates these previous findings by including more recently conducted studies, and includes additional reproductive factors (age at menarche, age at menopause, menopausal status, OC use, and HRT use) for evaluation. Consistent with these mechanisms, we observed that later age at menarche was associated with reduced risk of all four subtypes regardless of study design, although the association was weakest for luminal B and HER2-overexpressing BC. Post/peri-menopausal status was also associated with reduced BC risk for luminal A and luminal B, but not HER-2 overexpressing or TNBC in case–control studies. Misclassification of menopausal status may be possible here because most post-menopausal breast cancers are hormone-receptor positive. In case-only analysis, there was an increased risk of HER2-overexpressing and TNBC compared with luminal A breast cancer. Older age at menopause was associated with increased BC risk, with the strongest association seen in HER2-overexpressing BC. In the case-only analysis, younger vs. older age at menopause was associated with increased TNBC risk compared with luminal A [30, 59].

Higher parity was protective against BC risk regardless of study design, except for TNBC, where no significant association was observed across 27 studies. Lambertini et al. and Islami et al. observed that breastfeeding was associated with a lower risk of luminal and TNBC [6, 8], consistent with our findings showing significantly lower risk for luminal A, luminal B, HER2 + and TNBC subtypes with ever breastfeeding and with longer duration of breastfeeding. Breastfeeding is associated with additional prolonged reductions in estrogen levels locally in breast fluid by increased secretion of hormones like prolactin, glucocorticoids, and insulin [90]. Parous women experience reduced breast fluid estrogen due to the destruction of alveolar lobular breast tissue during post-partum [90]. Increased parity, younger age at first pregnancy, and lactation in breastfeeding women are found to be protective reproductive risk factors and restriction of these factors can cause prolonged exposure to endogenous estrogens and lead to breast cancer [90, 91]. We observed the strongest associations of OC use with TNBC subtype [23, 25, 37, 38]. In case-only studies, duration of OC use was associated with increased risk of HER2-overexpressing and TNBC, but not luminal B breast cancer [54, 76, 84, 89]. For HRT use, there was a strong association between duration of HRT use and ever use with all BC subtypes, [17, 19, 33, 36, 38, 42, 72], although the strongest association was observed in luminal A breast cancer [16, 17]. OC and HRT may drive BC risk via estrogen and/or progesterone-related pathways [92], as estrogens accelerate the mitotic rates of both normal and cancerous breast epithelial cells, and metabolites of estradiol are carcinogenic in this tissue type [93–95].

This review identified several gaps in the literature on luminal A, luminal B, HER2-overexpressing, and TNBC/basal-like subtypes and reproductive factors. History of abortion, pregnancy, and years since last birth could not be included in the meta-analysis due to an insufficient number of studies examining risk associations by molecular subtype. However, the existing studies indicate that women with a history of abortion have an increased risk of BC regardless of subtype, even though studies on abortion with BC subtypes are lacking [87]. Pregnancy has been shown to have a protective effect against BC; pregnancy has a shortterm increase in exposure to estrogen and progesterone, but overall reduces risk of breast cancer [93]. However, this also must be considered in relation to age at first pregnancy, because at later age of first pregnancy there is a higher probability of existing abnormal cells in the breast epithelium (due to accumulation of genomic errors with aging), and the burst of estrogens during pregnancy stimulates the growth of these pre-existing abnormal cells. This will then contribute to cancer progression in the breast tissue. Additional studies of subtype-specific risk associations for the history of abortion (spontaneous vs. induced), years since last pregnancy, and age at pregnancy at which abortion occurred are needed. Risk factors for breast cancer are thought to differ across menopausal status [96], however, due to the insufficient number of studies, a meta-analysis of reproductive risk factors by menopausal status could not be conducted. This review also reported that certain reproductive factors have a protective effect against BC; these factors include increased breastfeeding, higher parity and early age at first birth. We report that OC and HRT use have a high probability of increasing risk of BC and are non-protective risk factors. This review also includes a meta-analysis of eight reproductive risk factors, more than previously published systematic reviews and meta-analyses.

According to the American Cancer Society preventative guidelines, not having children, not breastfeeding, use of OC, and use of HRT after menopause increase the risk of breast cancer [97]. However, BC preventative guidelines lack information specific to molecular breast cancer subtypes, and breastfeeding information is not included in the most commonly used BC risk prediction model [98, 99]. This study demonstrates the potential utility of targeting interventions for modifiable reproductive factors such as breastfeeding to populations with high incidences of luminal A, luminal B, HER2-overexpressing, and TNBC/basal-like subtypes, which have a significant association with breastfeeding. Future studies might benefit from including reproductive risk factor associations combined with family history and other known risk factors to predict the risk of developing specific breast cancer subtypes.

Potential risk of biases of included studies should be considered. Meta-analytic associations between age at menarche, age at first birth, menopausal status, and parity with BC subtypes were often in opposing directions for case–control/cohort studies compared to case-case studies. Selection bias in the case–control/cohort studies is possible; additionally, interpretation of case-case studies has its limitations, as true risks relative to a cancer-free population cannot be directly estimated. Further, some of the results were unexpected in regards to the hypothesized associations in relation to biological mechanisms. For instance, hormone-dependent subtypes are more common in post-menopausal women, though we observed the opposite in case–control/cohort studies. Misclassification of menopausal status may be a contributing factor to these findings. Additionally, exposure to OC is expected to increase hormone-dependent BC, though we found a protective effect for luminal B in the case-case studies. Comprehensive data on OC formulation was not available for our analysis, though warrants further investigation to assess heterogeneity of effect by estrogen and progestin dosages. This review has several limitations: first, eligible studies were restricted to papers published in English, which may have excluded relevant studies from other populations. We did not conduct a formal risk of bias assessment, though we have discussed potential biases in the observed associations. Additionally, there were not enough studies examining pregnancy, abortion, and time since last birth associated with luminal breast cancer and TNBC subtypes, and we only included studies from the year 2000 to 2021. However, this study serves as the first comprehensive review and meta-analysis of the association between luminal A, luminal B, HER2-overexpressing, and TNBC subtypes and a wide range of reproductive factors.

## Conclusions

This study offers a comprehensive and up-to-date evaluation of the scientific literature on the associations between reproductive factors and luminal A, luminal B, HER2-overexpressing, and TNBC molecular subtypes. We identified a need for additional studies examining abortion as a risk factor and studies examining reproductive risk factors for BC subtypes stratified by patient menopausal status. Across all reproductive factors examined, age at menarche, age at first birth, parity, pregnancy, and breastfeeding showed relatively consistent risk associations across breast cancer subtypes. Considering this finding, common risk prediction models may be improved upon with the inclusion of breastfeeding status as a predictor.

## Supplementary Information

**Additional file 1:** **Supplemental Table 1.** Summary statistics for studies included after full-text review (*N*=75). **Supplemental Figure 1.** Age at menarche by molecular subtypes of Breast cancer. **Supplemental Figure 2.** Age at menarche by molecular subtypes of Breast cancer (Case vs Luminal A). **Supplemental Figure 3.** Age at menopause by molecular subtypes of Breast cancer. **Supplemental Figure 4.** Age at menopause by molecular subtypes of Breast cancer (Case vs Luminal A). **Supplemental Figure 5.** Menopausal status by molecular subtypes of Breast cancer. **Supplemental Figure 6.** Menopausal status by molecular subtypes of Breast cancer (Case vs Luminal A). **Supplemental Figure 7.** Age at first birth by molecular subtypes of Breast cancer. **Supplemental Figure 8.** Age at first birth by molecular subtypes of Breast cancer (Case vs Luminal A). **Supplemental Figure 9.** Parity by molecular subtypes of Breast cancer. **Supplemental Figure 10.** Parity by molecular subtypes of Breast cancer (Case vs Luminal A). **Supplemental Figure 11.** Breastfeeding by molecular subtypes of Breast cancer. **Supplemental Figure 12.** Breastfeeding by molecular subtypes of Breast cancer (Case vs Luminal A). **Supplemental Figure 13.** OC use by molecular subtypes of Breast cancer. **Supplemental Figure 14.** OC use by molecular subtypes of Breast cancer (Case vs Luminal A). **Supplemental Figure 15.** HRT use by molecular subtypes of Breast cancer. **Supplemental Figure 16.** HRT use by molecular subtypes of Breast cancer (Case vs Luminal A).

**Additional file 2.** Supplemental Appendix.

## Data Availability

Data were taken from publicly available publications and as such can be widely accessed. All data generated or analyzed during this study are included in this published article [and its supplementary information files]. The data used and/or analyzed during the current study are available from the corresponding author on reasonable request.
