# Supplementary material for: Association of reproductive risk factors and breast cancer molecular subtypes: a systematic review and meta-analysis
Source: BMC Cancer. 2023 Jul 10;23:644. doi: 10.1186/s12885-023-11049-0 (PMC10334550; doi:10.1186/s12885-023-11049-0)
Supplement: Supplementary file 1 — Additional file 1: Supplemental Table 1. Summary statistics for studies included after full-text review (N=75). Supplemental Figure 1. Age at menarche by molecular subtypes of Breast cancer. Supplemental Figure 2. Age at menarche by molecular subtypes of Breast cancer (Case vs Luminal A). Supplemental Figure 3. Age at menopause by molecular subtypes of Breast cancer. Supplemental Figure 4. Age at menopause by molecular subtypes of Breast cancer (Case vs Luminal A). Supplemental Figure 5. Menopausal status by molecular subtypes of Breast cancer. Supplemental Figure 6. Menopausal status by molecular subtypes of Breast cancer (Case vs Luminal A). Supplemental Figure 7. Age at first birth by molecular subtypes of Breast cancer. Supplemental Figure 8. Age at first birth by molecular subtypes of Breast cancer (Case vs Luminal A). Supplemental Figure 9. Parity by molecular subtypes of Breast cancer. Supplemental Figure 10. Parity by molecular subtypes of Breast cancer (Case vs Luminal A). Supplemental Figure 11. Breastfeeding by molecular subtypes of Breast cancer. Supplemental Figure 12. Breastfeeding by molecular subtypes of Breast cancer (Case vs Luminal A). Supplemental Figure 13. OC use by molecular subtypes of Breast cancer. Supplemental Figure 14. OC use by molecular subtypes of Breast cancer (Case vs Luminal A). Supplemental Figure 15. HRT use by molecular subtypes of Breast cancer. Supplemental Figure 16. HRT use by molecular subtypes of Breast cancer (Case vs Luminal A). [file 12885_2023_11049_MOESM1_ESM.docx]

**Supplemental Table 1. Summary statistics for studies included after full-text review (N=75)**

|  | **Total Analyzed Studies (n=75)** | **Luminal A**  **(n=32)** | **Luminal B**  **(n=38)** | **HER2-overexpressing**  **(n=62)** | **Triple negative/basal-like (n=65)** |
| --- | --- | --- | --- | --- | --- |
| **Publication Years** | | | | | |
| 2011-2021 | 59 | 26 | 32 | 49 | 54 |
| 2000-2010 | 16 | 6 | 6 | 13 | 11 |
| **Study Design** | | | | | |
| Case-Control studies | 40 | 21 | 18 | 32 | 35 |
| Case-Case studies | 26 | 7 | 16 | 20 | 23 |
| Cohort studies | 13 | 8 | 7 | 13 | 11 |
| **Region of study** | | | | | |
| USA | 35 | 17 | 16 | 27 | 35 |
| Asia | 15 | 11 | 12 | 15 | 13 |
| Europe | 15 | 13 | 12 | 15 | 13 |
| Multi-countries | 4 | 1 | 1 | 4 | 2 |
| Africa | 2 | 1 | 1 | 1 | 2 |
| Australia | 2 | 2 | 1 | 1 | 1 |
| Canada | 1 | 0 | 1 | 1 | 1 |
| Mexico | 3 | 1 | 1 | 3 | 2 |
| **Exposure of Interest** | | | | | |
| Pregnancy | 4 | 2 | 1 | 5 | 3 |
| Parity | 53 | 16 | 28 | 39 | 45 |
| Breastfeeding | 47 | 16 | 28 | 38 | 43 |
| Age at first birth | 44 | 17 | 25 | 37 | 40 |
| Years since last birth | 6 | 0 | 1 | 3 | 3 |
| Age at menarche | 46 | 26 | 24 | 35 | 37 |
| OC use | 23 | 7 | 15 | 20 | 21 |
| HRT use | 21 | 7 | 10 | 15 | 15 |
| Menopausal status | 18 | 7 | 11 | 16 | 18 |
| Age at menopause | 23 | 9 | 7 | 18 | 21 |
| Abortion | 5 | 3 | 3 | 6 | 4 |
| Abbreviations: HER2: Human epidermal growth factor receptor 2; OC: Oral Contraceptive; HRT: Hormone replacement therapy | | | | | |

**Supplemental Figure 1. Age at menarche by molecular subtypes of Breast cancer**

**Metabias: Begg:2.06, 0.039, Egger: -0.631, 0.004**

**Supplemental Figure 2. Age at menarche by molecular subtypes of Breast cancer (Case vs Luminal A)**


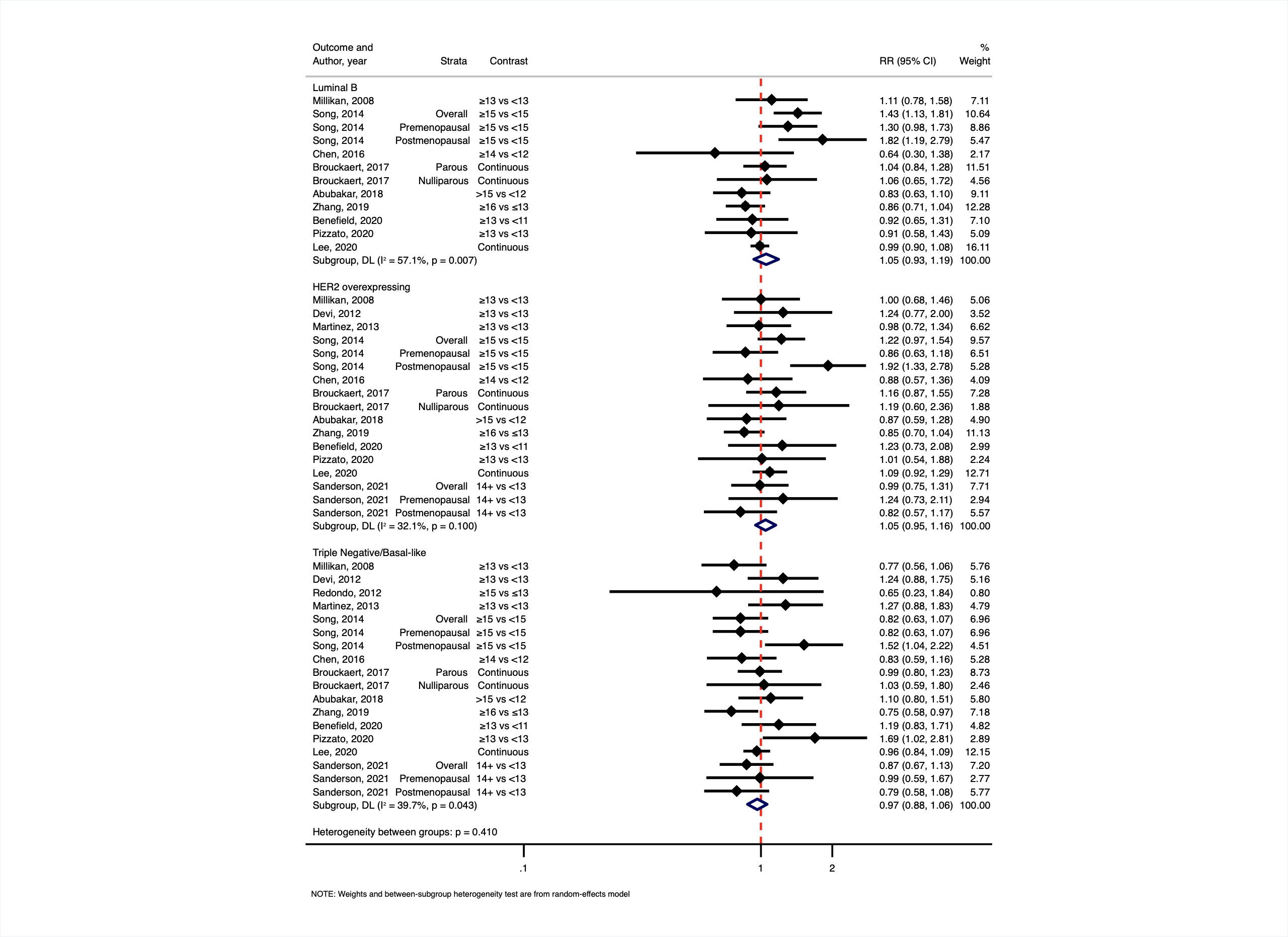


**Metabias: Begg: 1.48, p-value=0.140, Egger: 0.43, p=0.309**

**Supplemental Figure 3. Age at menopause by molecular subtypes of Breast cancer**

**Metabias: Begg 1.21, 0.227, Egger 0.615, 0.074**

**Supplemental Figure 4. Age at menopause by molecular subtypes of Breast cancer (Case vs Luminal A)**


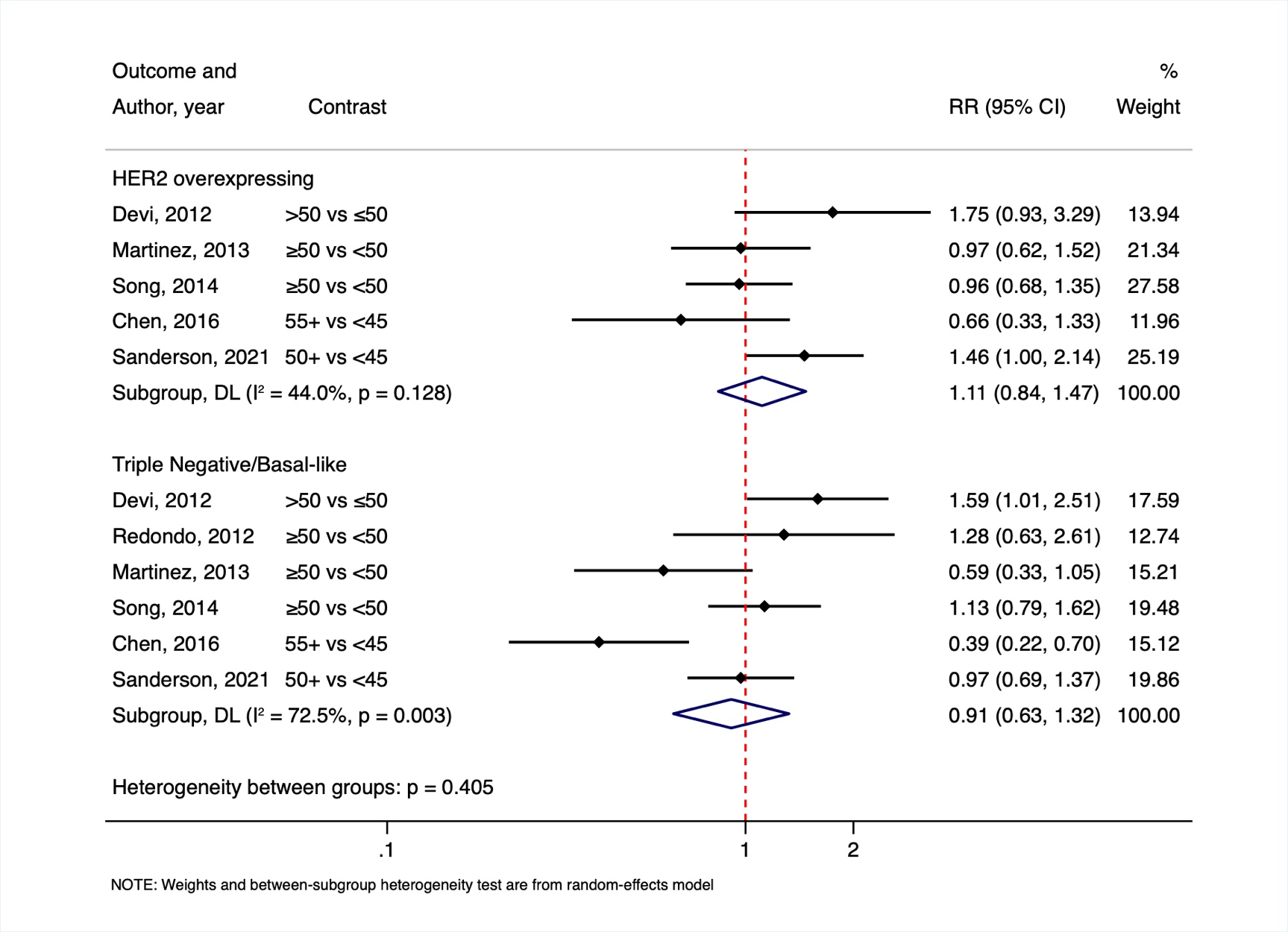


**Metabias: Begg: 0.01, p=0.991; Egger -1.04, p=0.06**

**Supplemental Figure 5. Menopausal status by molecular subtypes of Breast cancer**

**Metabias: Begg 2.25, p=0.025; 2.17, p <0.001**

**Supplemental Figure 6. Menopausal status by molecular subtypes of Breast cancer (Case vs Luminal A)**


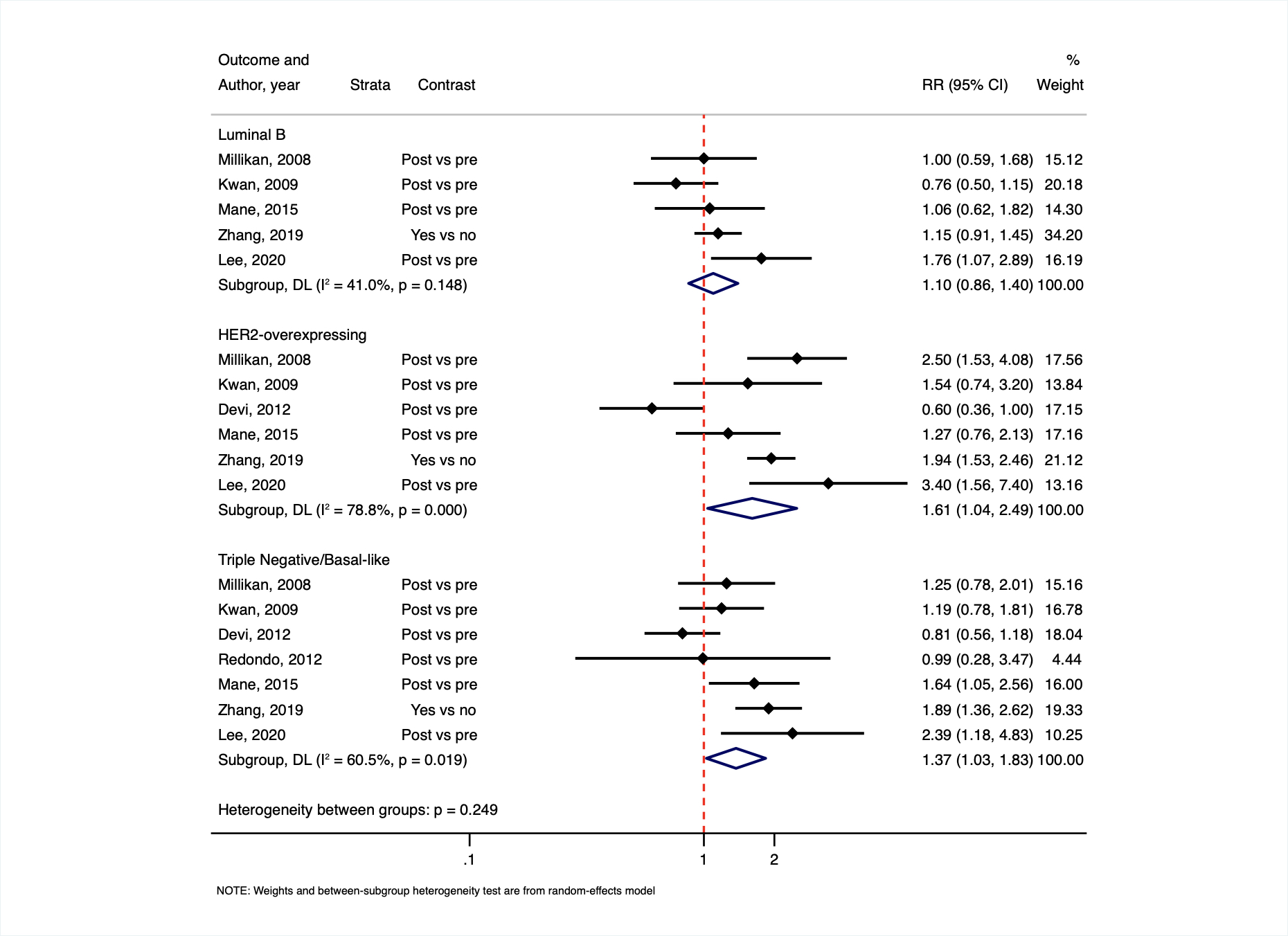


**Metabias: Begg: 0.677, p=0.705; Egger -0.138, p=0.910**

**Supplemental Figure 7. Age at first birth by molecular subtypes of Breast cancer**

**Metabias: Begg 1.92, 0.055; Egger 0.778, 0.001**

**Supplemental Figure 8. Age at first birth by molecular subtypes of Breast cancer (Case vs Luminal A)**


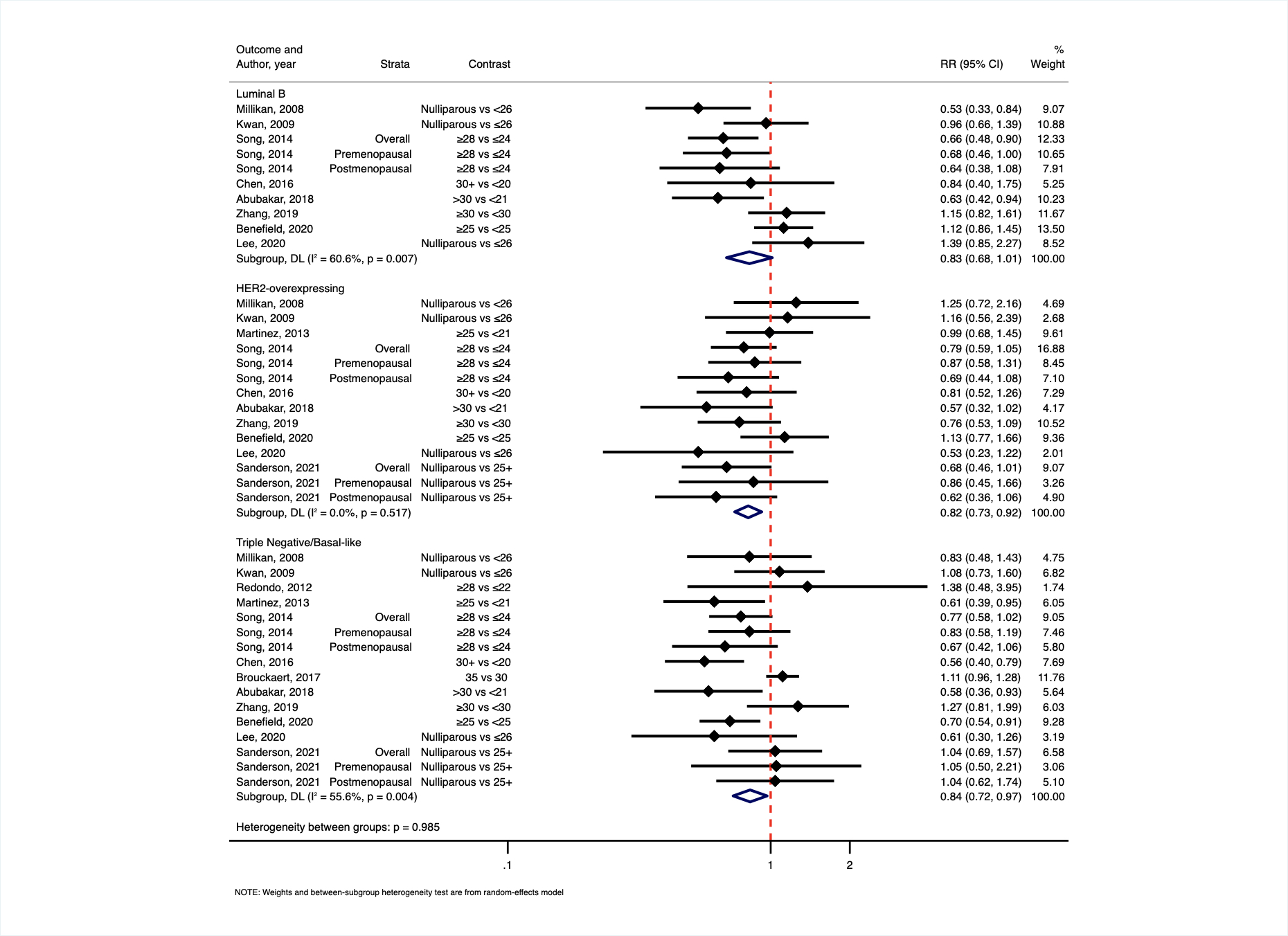


**Metabias: Begg: 0.01, p=0.991; Egger: -1.043, 0.059**

**Supplemental Figure 9. Parity by molecular subtypes of Breast cancer**

**Metabias: Egger .4656006, 0.304**

**Supplemental Figure 10. Parity by molecular subtypes of Breast cancer (Case vs Luminal A)**


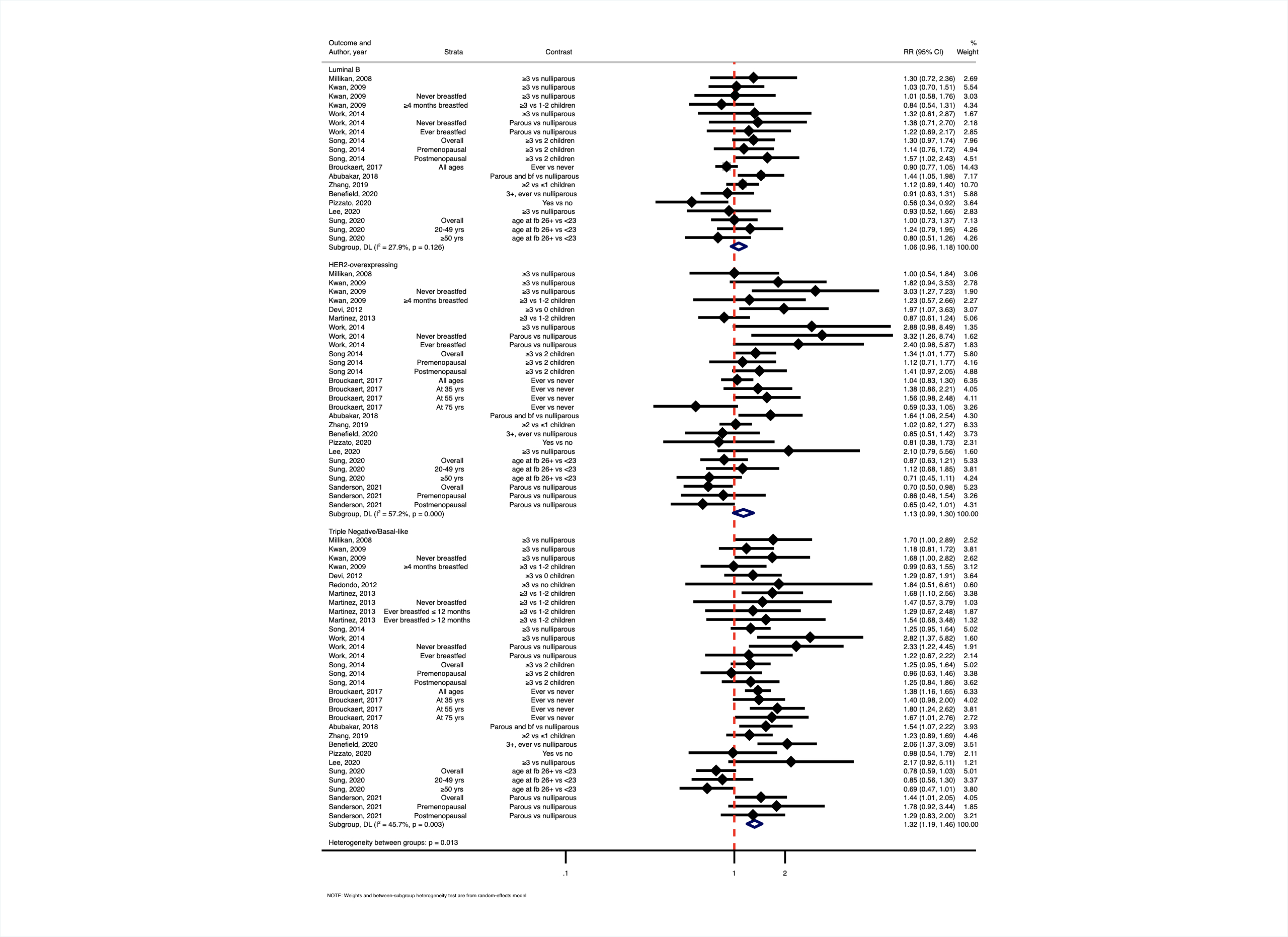


**Metabias: Begg: 2.47, p=0.013, Egger: 1.07, p=0.007**

**Supplemental Figure 11. Breastfeeding by molecular subtypes of Breast cancer**

**Metabias: 2.21, 0.027; Egger: -0.333, 0.499**

**Supplemental Figure 12. Breastfeeding by molecular subtypes of Breast cancer (Case vs Luminal A)**


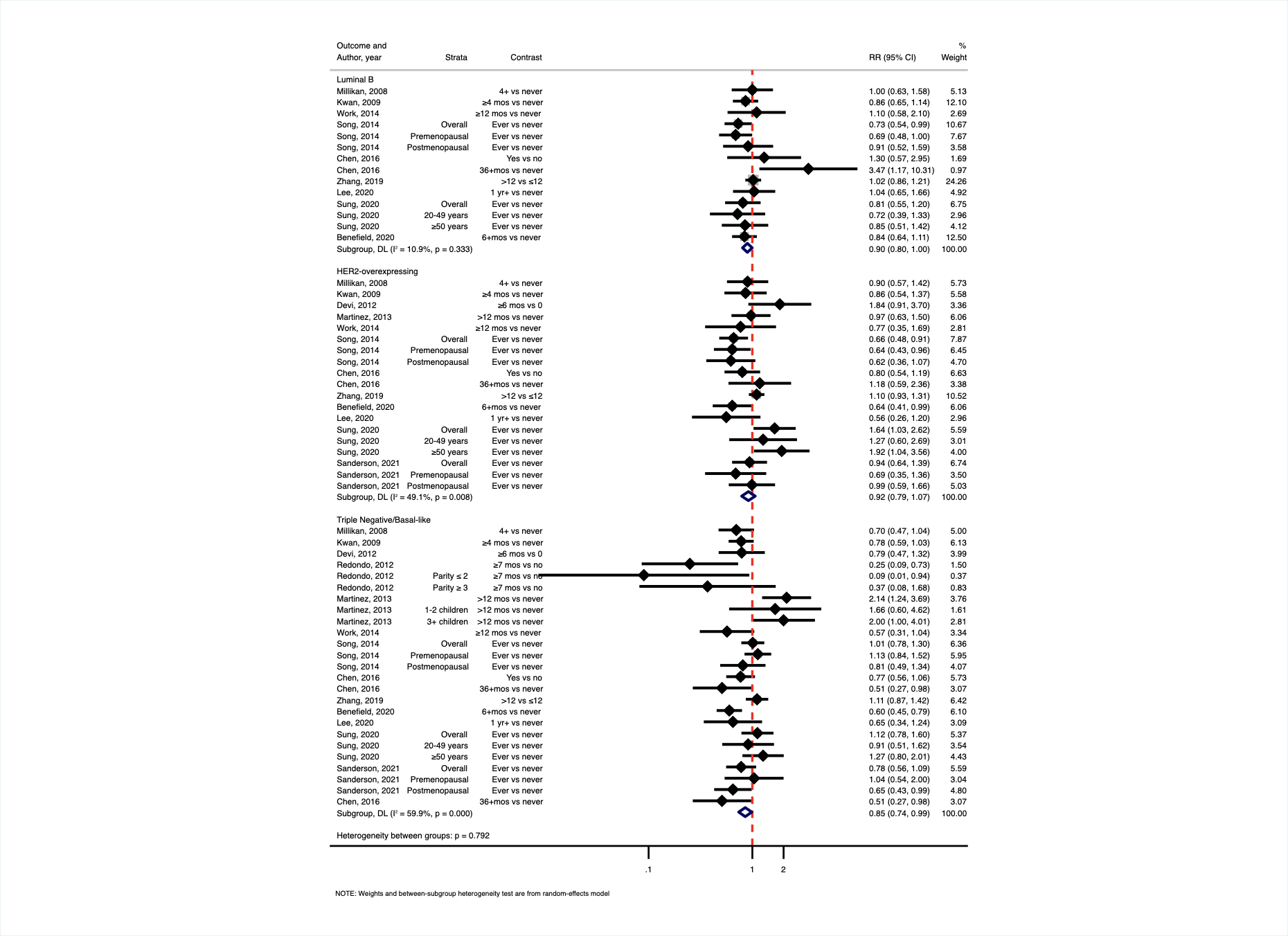


**Metabias: Begg: 0.38, p=0.705; Egger: -0.27, 0.529**

**Supplemental Figure 13. OC use by molecular subtypes of Breast cancer**

**Metabias: Begg 0.23, p=0.827, Egger .545, p=0.030**

**Supplemental Figure 14. OC use by molecular subtypes of Breast cancer (Case vs Luminal A)**


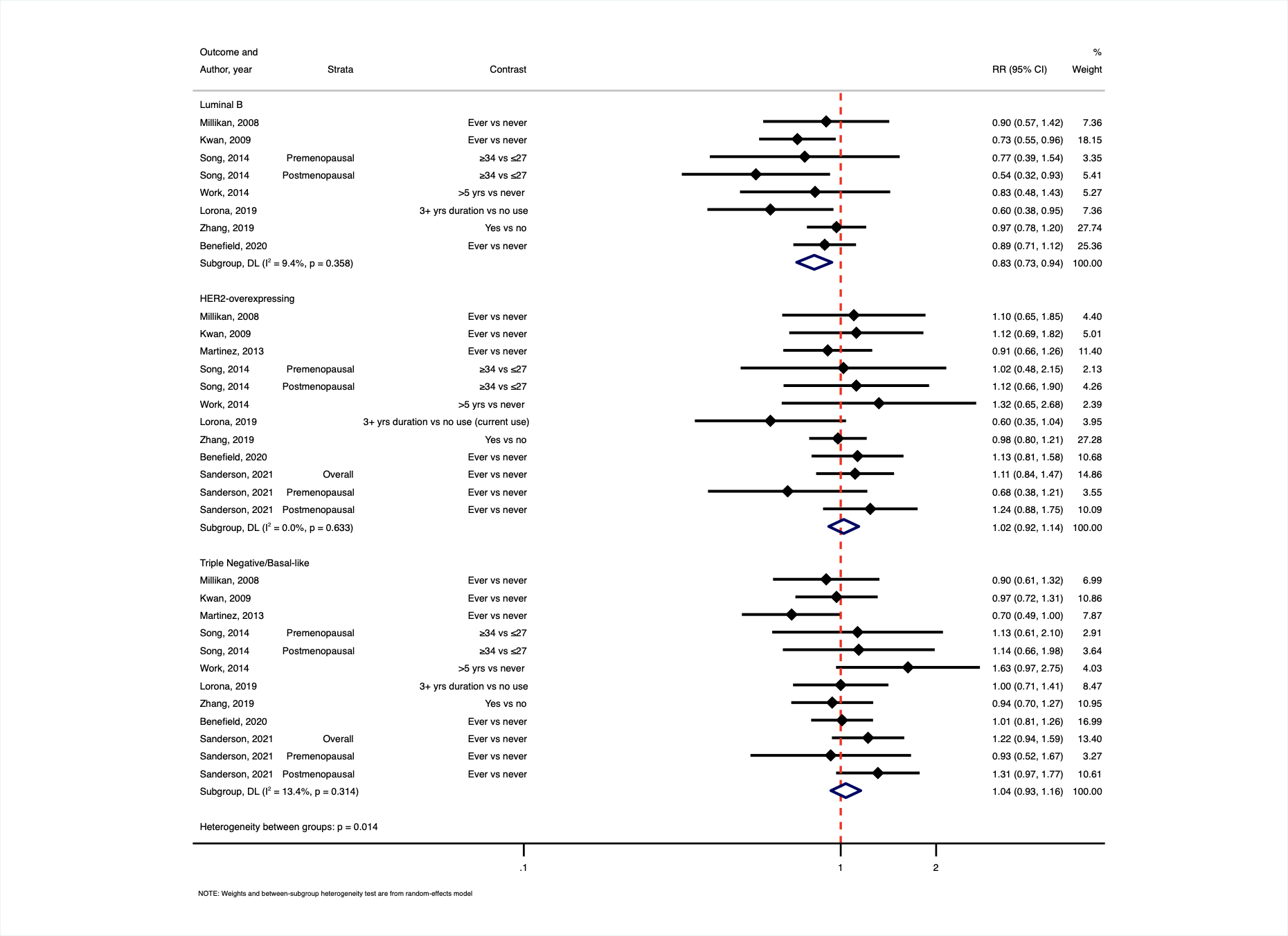


**Metabias: Begg: 0.47,0.638; Egger: -0.36, 0.527**

**Supplemental Figure 15. HRT use by molecular subtypes of Breast cancer.**

**Metabias: Begg 0.36, p=0.718; Egger -0.77, p=0.108**

**Supplemental Figure 16. HRT use by molecular subtypes of Breast cancer (Case vs Luminal A)**

**
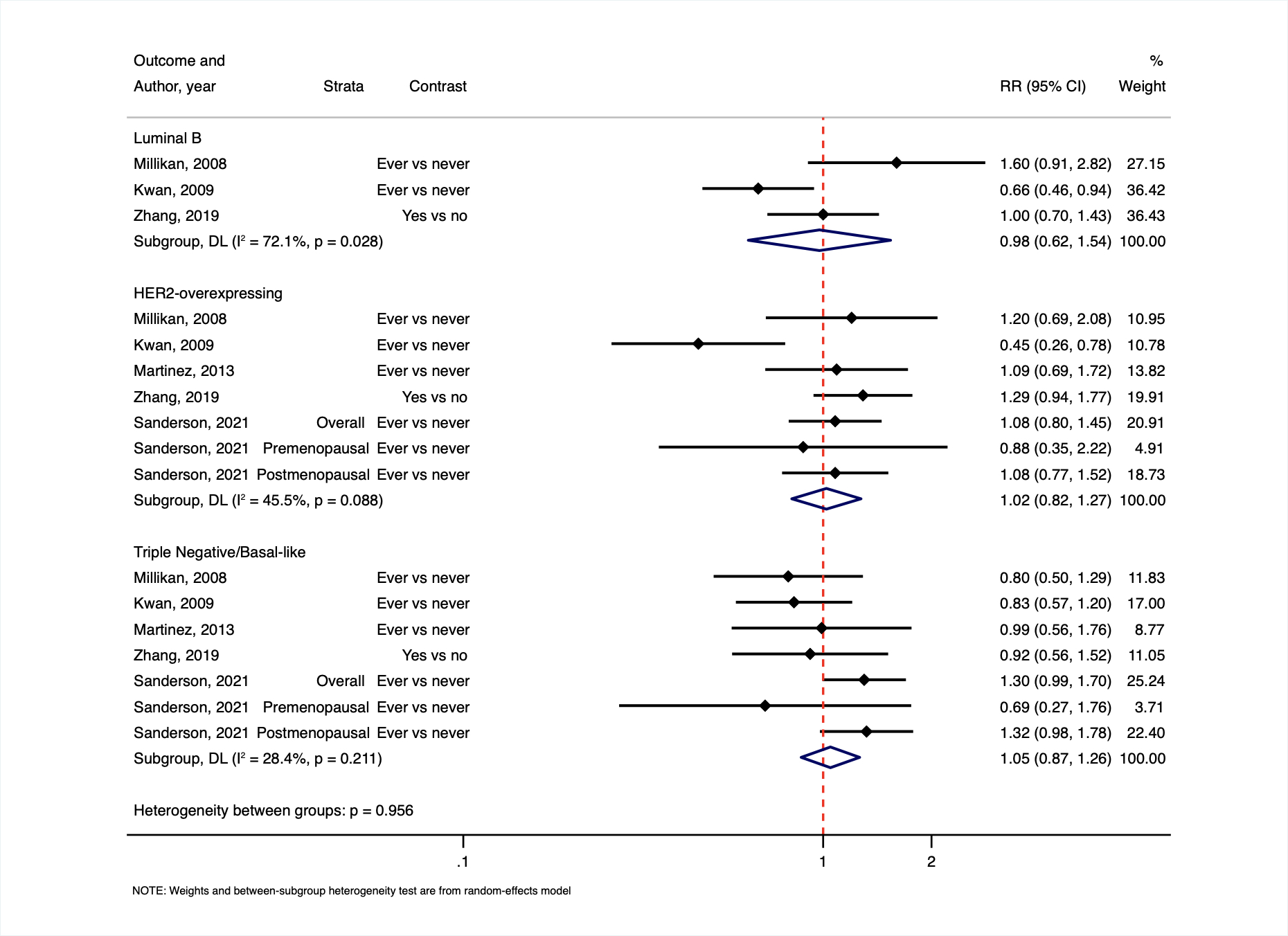
**

**Metabias: Begg: 1.85,p=0.064; Egger: -1.61, 0.123**
