# Supplementary material for: Association of reproductive risk factors and breast cancer molecular subtypes: a systematic review and meta-analysis
Source: BMC Cancer. 2023 Jul 10;23:644. doi: 10.1186/s12885-023-11049-0 (PMC10334550; doi:10.1186/s12885-023-11049-0)
Supplement: Supplementary file 2 — Additional file 2. Supplemental Appendix. [file 12885_2023_11049_MOESM2_ESM.docx]

**Supplemental Appendix**

**PubMed, Scopus, and Embase Search Strategy for Systematic Review**

1. ("Breast neoplasms"[Mesh:noexp] OR "Breast Carcinoma In Situ"[Mesh] OR "Carcinoma, Ductal, Breast"[Mesh] OR "Carcinoma, Lobular"[Mesh] OR "Hereditary Breast and Ovarian Cancer Syndrome"[Mesh] OR "Inflammatory Breast Neoplasms"[Mesh] OR "Triple Negative Breast Neoplasms"[Mesh] OR "Unilateral Breast Neoplasms"[Mesh] OR "breast neoplasm"[tiab] OR "breast neoplasms"[tiab] OR "breast malignant neoplasm"[tiab] OR "breast malignant neoplasms"[tiab] OR "breast tumor"[tiab] OR "breast tumors"[tiab] OR "breast cancer"[tiab] OR "breast cancers"[tiab] OR "cancer of breast"[tiab] OR "cancer of the breast"[tiab] OR "cancers of the breast"[tiab] OR "mammary carcinoma"[tiab] OR "mammary carcinomas"[tiab] OR "mammary neoplasm"[tiab] OR "mammary neoplasms"[tiab] OR "breast carcinoma"[tiab] OR "breast carcinomas"[tiab] OR "breast carcinogenesis"[tiab] OR "lobular carcinoma"[tiab] OR "lobular carcinomas"[tiab]) AND ("triple-negative"[tiab] OR "triple negative"[tiab] OR "Luminal A"[tiab] OR "Luminal B"[tiab] OR subtype[tiab] OR subtypes[tiab] OR "basal-like"[tiab] OR "basal like"[tiab] OR "HER2"[tiab] OR "HER 2"[tiab] OR "HER-2"[tiab] OR "HER2/neu"[tiab] OR "HER 2/neu"[tiab] OR "HER-2/neu"[tiab] OR "human epidermal growth factor receptor 2"[tiab] OR "human epidermal growth factor receptor-2"[tiab])

"Reproductive behavior"[Mesh] OR "Abortion, spontaneous"[Mesh]

OR "Reproductive history"[Mesh] OR Parity[Mesh] OR "Menarche"[Mesh] OR "Maternal age"[Mesh] OR "Breast Feeding"[Mesh] OR "Pregnancy"[Mesh] OR "Contraceptives, oral"[Mesh] OR "Abortion, induced"[Mesh] OR "reproductive behavior"[tiab] OR "reproductive behaviors"[tiab] OR "reproductive history"[tiab] OR "reproductive histories"[tiab] OR "reproductive factor"[tiab] OR "reproductive factors"[tiab] OR "reproductive characteristic"[tiab] OR "reproductive characteristics"[tiab] OR "reproductive experience"[tiab] OR "reproductive experiences"[tiab] OR "reproductive risk factor"[tiab] OR "reproductive risk factors"[tiab] OR "maternal age"[tiab] OR parity[tiab] OR parous[tiab] OR multiparous[tiab] OR "first birth"[tiab] OR "last birth"[tiab] OR breastfeeding[tiab] OR "breast feeding"[tiab] OR menarche[tiab] OR menopause[tiab] OR menopausal[tiab] OR premenopause[tiab] OR premenopausal[tiab] OR postmenopause[tiab] OR postmenopausal[tiab] OR pregnancy[tiab] OR pregnancies[tiab] OR "HRT"[tiab] OR "hormone replacement"[tiab] OR "oral contraceptive"[tiab] OR "oral contraceptives"[tiab] OR abortion[tiab] OR abortions[tiab] OR miscarriage[tiab] OR miscarriages[tiab]

1. ('Breast cancer'/de OR 'Breast Carcinoma'/exp OR 'Lobular carcinoma'/exp OR 'Hereditary Breast and Ovarian Cancer Syndrome'/exp OR 'Inflammatory Breast cancer'/exp OR 'Triple Negative Breast cancer'/exp OR 'Unilateral Breast cancer'/exp OR 'breast neoplasm':ab,ti OR 'breast neoplasms':ab,ti OR 'breast malignant neoplasm':ab,ti OR 'breast malignant neoplasms':ab,ti OR 'breast tumor':ab,ti OR 'breast tumors':ab,ti OR 'breast cancer':ab,ti OR 'breast cancers':ab,ti OR 'cancer of breast':ab,ti OR 'cancer of the breast':ab,ti OR 'cancers of the breast':ab,ti OR 'mammary carcinoma':ab,ti OR 'mammary carcinomas':ab,ti OR 'mammary neoplasm':ab,ti OR 'mammary neoplasms':ab,ti OR 'breast carcinoma':ab,ti OR 'breast carcinomas':ab,ti OR 'breast carcinogenesis':ab,ti OR 'lobular carcinoma':ab,ti OR 'lobular carcinomas':ab,ti) AND ('triple-negative':ab,ti OR 'triple negative':ab,ti OR 'Luminal A':ab,ti OR 'Luminal B':ab,ti OR subtype:ab,ti OR subtypes:ab,ti OR 'basal-like':ab,ti OR 'basal like':ab,ti OR 'HER2':ab,ti OR 'HER 2':ab,ti OR 'HER-2':ab,ti OR 'HER2/neu':ab,ti OR 'HER 2/neu':ab,ti OR 'HER-2/neu':ab,ti OR 'human epidermal growth factor receptor 2':ab,ti OR 'human epidermal growth factor receptor-2':ab,ti) 'Reproductive behavior'/exp OR 'Abortion'/exp

OR 'Reproductive history'/exp OR Parity/exp OR 'Menarche'/exp OR 'Maternal age'/exp OR 'Breast Feeding'/exp OR 'Pregnancy'/exp OR 'Oral contraceptive agent'/exp OR 'Induced abortion'/exp OR 'reproductive behavior':ab,ti OR 'reproductive behaviors':ab,ti OR 'reproductive history':ab,ti OR 'reproductive histories':ab,ti OR 'reproductive factor':ab,ti OR 'reproductive factors':ab,ti OR 'reproductive characteristic':ab,ti OR 'reproductive characteristics':ab,ti OR 'reproductive experience':ab,ti OR 'reproductive experiences':ab,ti OR 'reproductive risk factor':ab,ti OR 'reproductive risk factors':ab,ti OR 'maternal age':ab,ti OR parity:ab,ti OR parous:ab,ti OR multiparous:ab,ti OR 'first birth':ab,ti OR 'last birth':ab,ti OR breastfeeding:ab,ti OR 'breast feeding':ab,ti OR menarche:ab,ti OR menopause:ab,ti OR menopausal:ab,ti OR premenopause:ab,ti OR premenopausal:ab,ti OR postmenopause:ab,ti OR postmenopausal:ab,ti OR pregnancy:ab,ti OR pregnancies:ab,ti OR 'HRT':ab,ti OR 'hormone replacement':ab,ti OR 'oral contraceptive':ab,ti OR 'oral contraceptives':ab,ti OR abortion:ab,ti OR abortions:ab,ti OR miscarriage:ab,ti OR miscarriages:ab,ti

1. TS=(("breast neoplasm" OR "breast neoplasms" OR "breast malignant neoplasm" OR "breast malignant neoplasms" OR "breast tumor" OR "breast tumors" OR "breast cancer" OR "breast cancers" OR "cancer of breast" OR "cancer of the breast" OR "cancers of the breast" OR "mammary carcinoma" OR "mammary carcinomas" OR "mammary neoplasm" OR "mammary neoplasms" OR "breast carcinoma" OR "breast carcinomas" OR "breast carcinogenesis" OR "lobular carcinoma" OR "lobular carcinomas") AND ("triple-negative" OR "triple negative" OR "Luminal A" OR "Luminal B" OR subtype OR subtypes OR "basal-like" OR "basal like" OR "HER2" OR "HER 2" OR "HER-2" OR "HER2/neu" OR "HER 2/neu" OR "HER-2/neu" OR "human epidermal growth factor receptor 2" OR "human epidermal growth factor receptor-2")) TS=( "reproductive behavior" OR "reproductive behaviors" OR "reproductive history" OR "reproductive histories" OR "reproductive factor" OR "reproductive factors" OR "reproductive characteristic" OR "reproductive characteristics" OR "reproductive experience" OR "reproductive experiences" OR "reproductive risk factor" OR "reproductive risk factors" OR "maternal age" OR parity OR parous OR multiparous OR "first birth" OR "last birth" OR breastfeeding OR "breast feeding" OR menarche OR menopause OR menopausal OR premenopause OR premenopausal OR postmenopause OR postmenopausal OR pregnancy OR pregnancies OR "HRT" OR "hormone replacement" OR "oral contraceptive" OR "oral contraceptives" OR abortion OR abortions OR miscarriage OR miscarriages)
